# Supplementary material for: Selenium Status in Patients with Chronic Liver Disease: A Systematic Review and Meta-Analysis
Source: Nutrients. 2022 Feb 23;14(5):952. doi: 10.3390/nu14050952 (PMC8912406; doi:10.3390/nu14050952)
Supplement: Supplementary file 1 [file nutrients-14-00952-s001.zip › supplementary material revised.pdf]

Selenium status in patients with liver disease: a systematic review and meta-analysis

Yaduan Lin et al.

Online Supplemental Material

Study	SMD (95% CI)	Weight %
Shamberger RJ 1973	-2.71 (-3.71, -1.72)	0.73
Shamberger RJ 1973	-2.42 (-3.69, -1.16)	0.71
Shamberger RJ 1973	-2.36 (-3.09, -1.63)	0.74
Burch RE 1978	-2.76 (-4.00, -1.52)	0.72
Sullivan JF 1979	-2.24 (-3.00, -1.48)	0.73
Aaseth J 1980	-3.71 (-4.70, -2.71)	0.73
Aaseth J 1982	-1.02 (-1.71, -0.32)	0.74
Aaseth J 1982	-1.19 (-2.25, -0.13)	0.72
Aaseth J 1982	-1.22 (-1.91, -0.53)	0.74
Aaseth J 1982	-0.67 (-1.33, -0.01)	0.74
Aaseth J 1982	-1.61 (-2.28, -0.94)	0.74
Välimäki MJ 1983	-2.61 (-3.40, -1.83)	0.73
Välimäki MJ 1983	-2.55 (-3.27, -1.83)	0.74
Välimäki MJ 1983	-0.98 (-1.46, -0.51)	0.74
Korpela H 1985	-4.01 (-5.02, -3.00)	0.73
Korpela H 1985	-3.68 (-4.98, -2.38)	0.71
Korpela H 1985	-2.23 (-3.12, -1.34)	0.73
Corrocher R 1986	-1.62 (-2.66, -0.58)	0.72
Johansson U 1986	-1.93 (-2.72, -1.14)	0.73
Johansson U 1986	-1.66 (-2.42, -0.90)	0.73
Johansson U 1986	-0.10 (-0.81, 0.60)	0.74
Sharda B 1986	6.00 (4.52, 7.48)	0.70
Sharda B 1986	-1.41 (-2.11, -0.72)	0.74
Sharda B 1986	6.63 (5.01, 8.24)	0.70
Milman N 1986	-5.14 (-6.09, -4.18)	0.73
Milman N 1986	15.91 (13.45, 18.38)	0.64
Dworkin B. M 1988	-4.41 (-5.90, -2.91)	0.70
Casaril, M. 1989	-1.92 (-2.39, -1.45)	0.74
Välimäki M 1991	-1.80 (-2.99, -0.62)	0.72
Gerli G 1992	-0.52 (-1.03, -0.01)	0.74
Gerli G 1992	-1.12 (-1.60, -0.63)	0.74
Gerli G 1992	-1.94 (-2.55, -1.34)	0.74
Gerli G 1992	-0.49 (-1.01, 0.02)	0.74
Thuluvath PJ 1992	-1.39 (-2.17, -0.61)	0.73
Thuluvath PJ 1992	-1.48 (-1.92, -1.03)	0.74
Thuluvath PJ 1992	-1.22 (-1.63, -0.81)	0.74
Thuluvath PJ 1992	-1.07 (-1.80, -0.34)	0.74
Thuluvath PJ 1992	-0.53 (-1.08, 0.02)	0.74
Thuluvath PJ 1992	-0.37 (-0.82, 0.09)	0.74
Ward R J 1992	-0.76 (-1.65, 0.13)	0.73
Ward R J 1992	-1.46 (-2.67, -0.25)	0.72
Ward R J 1992	-1.58 (-2.90, -0.26)	0.71
Buljevac, M. 1996	-2.70 (-3.38, -2.01)	0.74
Buljevac, M. 1996	-2.68 (-3.08, -2.29)	0.74
Loguercio C 1997	0.94 (-0.04, 1.93)	0.73
Loguercio C 1997	-1.53 (-2.60, -0.46)	0.72
Loguercio C 1997	-4.00 (-5.43, -2.57)	0.71
Loguercio C 1997	-3.00 (-4.12, -1.88)	0.72
Loguercio C 1997	-4.35 (-5.88, -2.83)	0.70
Loguercio C 1997	-4.12 (-5.48, -2.76)	0.71
Guarini P 1998	-0.95 (-1.60, -0.29)	0.74
Guarini P 1998	-1.22 (-1.80, -0.64)	0.74
Yu MW 1999	-0.15 (-0.38, 0.09)	0.75
Yu MW 1999	-0.55 (-0.84, -0.26)	0.74
Chin-Thin W 2002	-2.25 (-2.75, -1.75)	0.74
Navarro-Alarcón, M 2002	-0.89 (-1.27, -0.52)	0.74
Navarro-Alarcón, M 2002	-1.28 (-1.89, -0.67)	0.74
Czuczejko J 2003	-2.07 (-2.67, -1.47)	0.74
Czuczejko J 2003	-0.99 (-1.44, -0.54)	0.74
Czuczejko J 2003	-2.01 (-2.61, -1.42)	0.74
Czuczejko J 2003	-1.18 (-1.64, -0.71)	0.74
Czuczejko J 2003	-2.37 (-2.97, -1.76)	0.74
Czuczejko J 2003	-1.53 (-2.01, -1.05)	0.74
Czuczejko J 2003	-2.57 (-3.20, -1.94)	0.74
Czuczejko J 2003	-1.41 (-1.89, -0.94)	0.74
Sayed, H. A. 2005	-0.61 (-1.12, -0.10)	0.74
Sayed, H. A. 2005	-0.57 (-1.22, 0.09)	0.74
Sayed, H. A. 2005	-0.36 (-1.22, 0.50)	0.73
Ko, W.S. 2005	-8.96 (-10.61, -7.31)	0.69
Ko, W.S. 2005	-17.34 (-20.43, -14.25)	0.59
Pemberton PW 2005	-11.09 (-12.98, -9.20)	0.68
Sakoda LC 2005	-0.45 (-0.63, -0.26)	0.75
Lin CC 2006	-0.26 (-0.68, 0.16)	0.74
Lin CC 2006	-0.21 (-0.61, 0.18)	0.74
Lin CC 2006	-0.95 (-1.51, -0.39)	0.74
Lin CC 2006	-0.51 (-1.03, 0.02)	0.74
Váli L 2008	0.19 (-0.50, 0.88)	0.74
Váli L 2008	-2.99 (-3.79, -2.20)	0.73
González-Reimers E 2009	-0.09 (-0.87, 0.70)	0.73
González-Reimers E 2009	-0.76 (-1.51, -0.02)	0.74
González-Reimers E 2009	-1.03 (-1.70, -0.36)	0.74
Balamtekin, N. 2010	-2.76 (-3.46, -2.05)	0.74
Uslu, N. 2010	-1.64 (-2.15, -1.13)	0.74
Martínez-Peinado M 2010	-0.86 (-1.28, -0.43)	0.74
Himoto, T. 2011	-1.14 (-2.07, -0.21)	0.73
Himoto, T. 2011	-2.39 (-3.53, -1.24)	0.72
Kazi, TG 2012	-3.35 (-3.89, -2.80)	0.74
Kazi, TG 2012	-15.80 (-17.64, -13.96)	0.68
Kazi, TG 2012	-2.66 (-3.11, -2.20)	0.74
Kazi, TG 2012	-9.16 (-10.22, -8.09)	0.72
Kolachi NF 2012	-1.49 (-2.02, -0.96)	0.74
Kolachi NF 2012	-4.95 (-5.89, -4.01)	0.73
Kolachi NF 2012	-9.26 (-10.86, -7.65)	0.70
Kolachi NF 2012	-3.14 (-3.75, -2.53)	0.74
Kolachi NF 2012	-3.17 (-3.78, -2.56)	0.74
Kolachi NF 2012	-9.29 (-10.68, -7.89)	0.71
Kolachi NF 2012	-1.37 (-2.06, -0.68)	0.74
Kolachi NF 2012	-3.35 (-4.29, -2.41)	0.73
Kolachi NF 2012	-7.36 (-9.02, -5.70)	0.69
Kolachi NF 2012	-2.19 (-2.80, -1.58)	0.74
Kolachi NF 2012	-2.15 (-2.76, -1.54)	0.74
Kolachi NF 2012	-8.69 (-10.23, -7.14)	0.70
Kim IW 2012	-0.53 (-0.79, -0.27)	0.75
Kim IW 2012	-0.38 (-1.03, 0.27)	0.74
Kim IW 2012	-1.50 (-1.94, -1.07)	0.74
Kim IW 2012	-0.40 (-0.80, 0.01)	0.74
Khan MS 2012	-0.71 (-1.81, 0.40)	0.72
Khan MS 2012	-2.59 (-3.47, -1.70)	0.73
Khan MS 2012	-1.21 (-1.84, -0.58)	0.74
Khan MS 2012	-1.64 (-2.23, -1.04)	0.74
Kolachi, NF. 2013	-11.83 (-12.87, -10.78)	0.72
Kolachi, NF. 2013	-3.58 (-3.97, -3.19)	0.74
Pan D 2013	-0.68 (-1.43, 0.07)	0.73
Pan D 2013	-0.39 (-0.97, 0.19)	0.74
Pan D 2013	-0.73 (-1.69, 0.24)	0.73
Pan D 2013	-0.26 (-1.42, 0.91)	0.72
Pan D 2013	-0.88 (-1.71, -0.04)	0.73
Pan D 2013	-1.44 (-2.22, -0.67)	0.73
Pan D 2013	-1.02 (-1.55, -0.50)	0.74
Pan D 2013	-1.90 (-2.72, -1.08)	0.73
Bettinger, D. 2013	-2.04 (-3.09, -0.99)	0.72
Bettinger, D. 2013	-2.36 (-3.53, -1.19)	0.72
Bettinger, D. 2013	-1.31 (-2.28, -0.33)	0.73
Nangliya V 2015	-3.45 (-3.91, -2.98)	0.74
Yang Z 2016	17.57 (17.30, 17.83)	0.75
Hughes DJ 2016	-0.31 (-0.75, 0.13)	0.74
Hughes DJ 2016	-0.42 (-0.70, -0.14)	0.74
Hughes DJ 2016	-1.66 (-1.95, -1.36)	0.74
Prystupa A 2017	-2.35 (-3.09, -1.61)	0.74
Prystupa A 2017	-1.00 (-1.58, -0.42)	0.74
Prystupa A 2017	-1.79 (-2.44, -1.14)	0.74
Li L 2020	-0.20 (-0.39, -0.01)	0.75
Li L 2020	0.15 (0.03, 0.27)	0.75
Fu L 2020	-2.53 (-2.89, -2.16)	0.74
Dhanda A 2020	-2.36 (-2.91, -1.81)	0.74
Dhanda A 2020	-1.67 (-2.58, -0.76)	0.73
Wang X 2021	0.06 (-0.01, 0.12)	0.75
Overall (I^2 = 99.4%, p = 0.000)	-1.90 (-2.42, -1.39)	100.00

NOTE: Weights are from random effects analysis

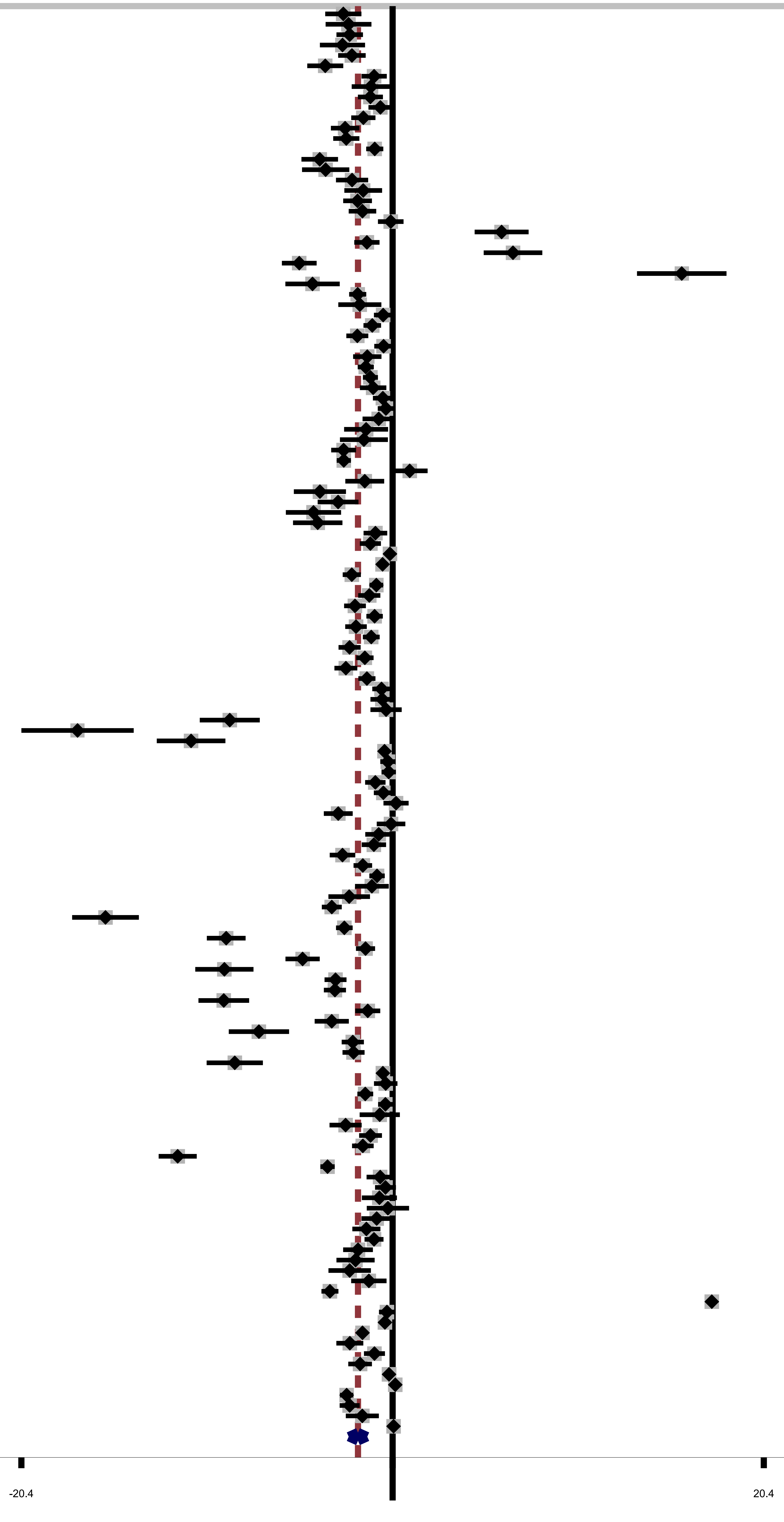

Figure S1. Forest plot in the meta-analysis of selenium levels and overall chronic liver diseases. The dot represents the effect size of each study. The line through the dot represents 95% CI of the effect size[13,16,17,19,30,32-77]. The size of the square represents the weight, Wi. The broken line represents the effect size and the horizontal length of the diamond the 95% CI of the effect size for all studies combined.

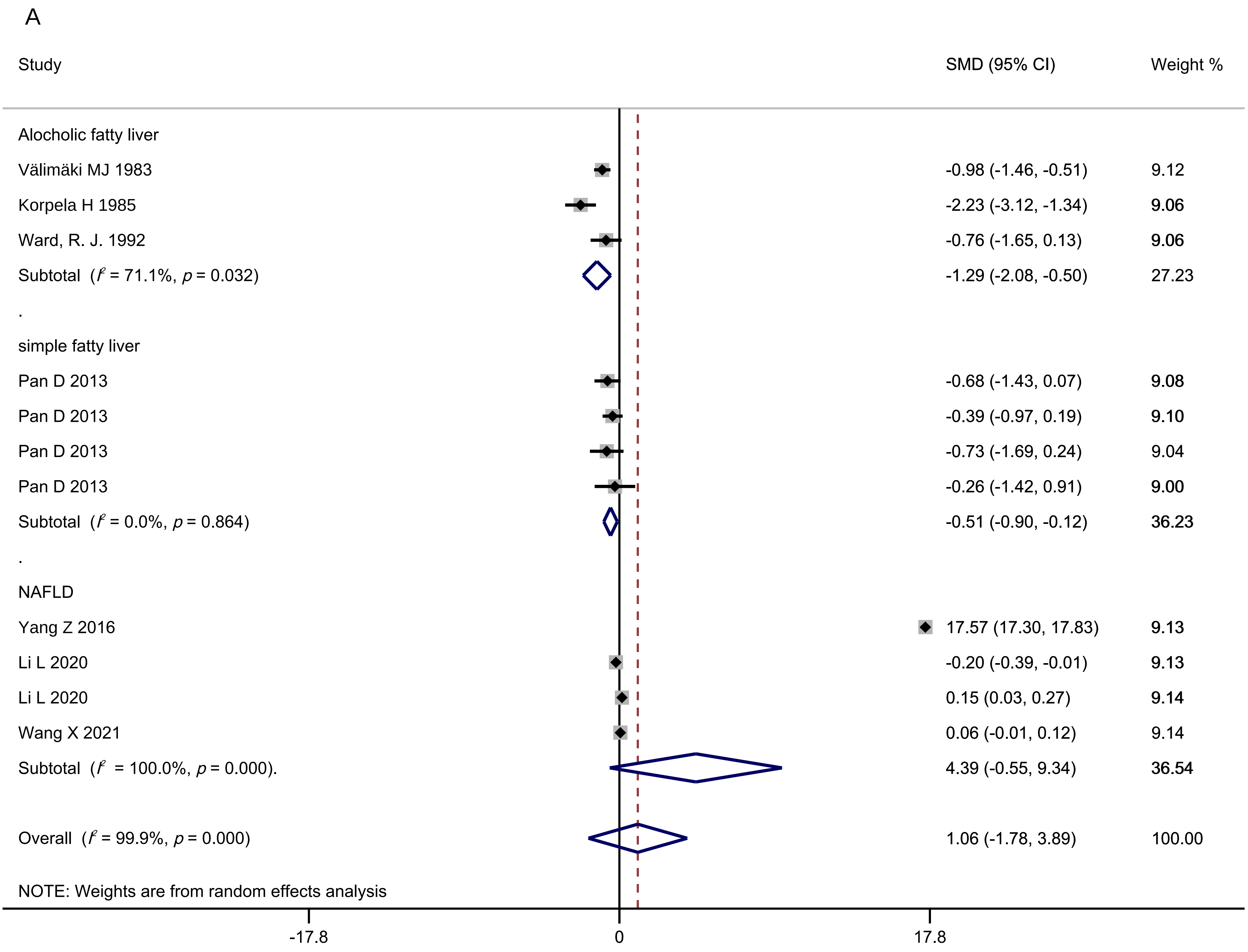

A
Study
SMD (95% CI)
Weight %
Alocholic fatty liver
Välimäki MJ 1983 -0.98 (-1.46, -0.51) 9.12
Korpela H 1985 -2.23 (-3.12, -1.34) 9.06
Ward, R. J. 1992 -0.76 (-1.65, 0.13) 9.06
Subtotal (I² = 71.1%, p = 0.032) -1.29 (-2.08, -0.50) 27.23
.
simple fatty liver
Pan D 2013 -0.68 (-1.43, 0.07) 9.08
Pan D 2013 -0.39 (-0.97, 0.19) 9.10
Pan D 2013 -0.73 (-1.69, 0.24) 9.04
Pan D 2013 -0.26 (-1.42, 0.91) 9.00
Subtotal (I² = 0.0%, p = 0.864) -0.51 (-0.90, -0.12) 36.23
.
NAFLD
Yang Z 2016 17.57 (17.30, 17.83) 9.13
Li L 2020 -0.20 (-0.39, -0.01) 9.13
Li L 2020 0.15 (0.03, 0.27) 9.14
Wang X 2021 0.06 (-0.01, 0.12) 9.14
Subtotal (I² = 100.0%, p = 0.000). 4.39 (-0.55, 9.34) 36.54
Overall (I² = 99.9%, p = 0.000) 1.06 (-1.78, 3.89) 100.00
NOTE: Weights are from random effects analysis
-17.8
0
17.8

B

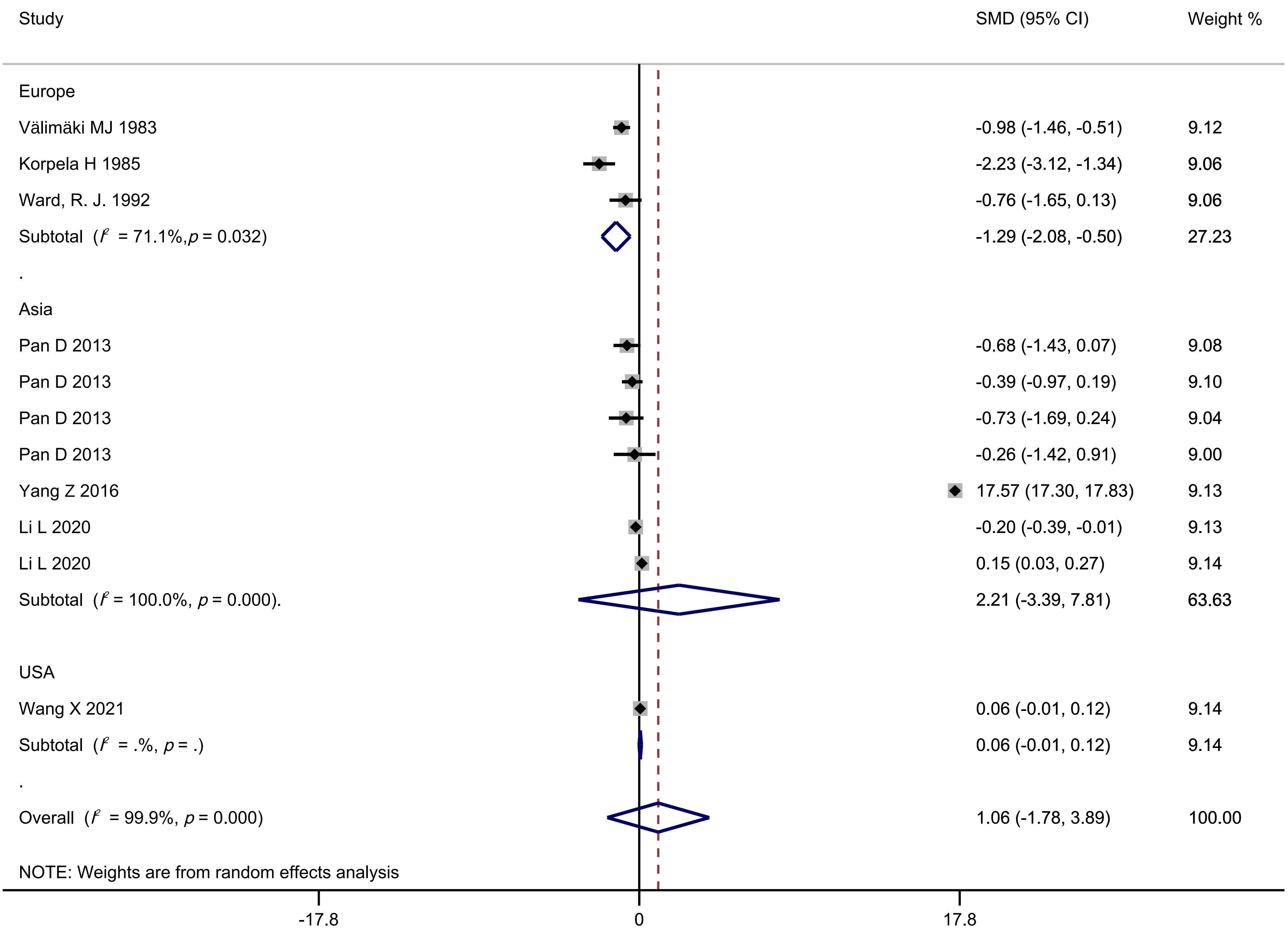

Study
SMD (95% CI)
Weight %
Europe
Välimäki MJ 1983 -0.98 (-1.46, -0.51) 9.12
Korpela H 1985 -2.23 (-3.12, -1.34) 9.06
Ward, R. J. 1992 -0.76 (-1.65, 0.13) 9.06
Subtotal (I² = 71.1%, p = 0.032) -1.29 (-2.08, -0.50) 27.23
.
Asia
Pan D 2013 -0.68 (-1.43, 0.07) 9.08
Pan D 2013 -0.39 (-0.97, 0.19) 9.10
Pan D 2013 -0.73 (-1.69, 0.24) 9.04
Pan D 2013 -0.26 (-1.42, 0.91) 9.00
Yang Z 2016 17.57 (17.30, 17.83) 9.13
Li L 2020 -0.20 (-0.39, -0.01) 9.13
Li L 2020 0.15 (0.03, 0.27) 9.14
Subtotal (I² = 100.0%, p = 0.000). 2.21 (-3.39, 7.81) 63.63
USA
Wang X 2021 0.06 (-0.01, 0.12) 9.14
Subtotal (I² = .%, p = .) 0.06 (-0.01, 0.12) 9.14
.
Overall (I² = 99.9%, p = 0.000) 1.06 (-1.78, 3.89) 100.00
NOTE: Weights are from random effects analysis
-17.8
0
17.8

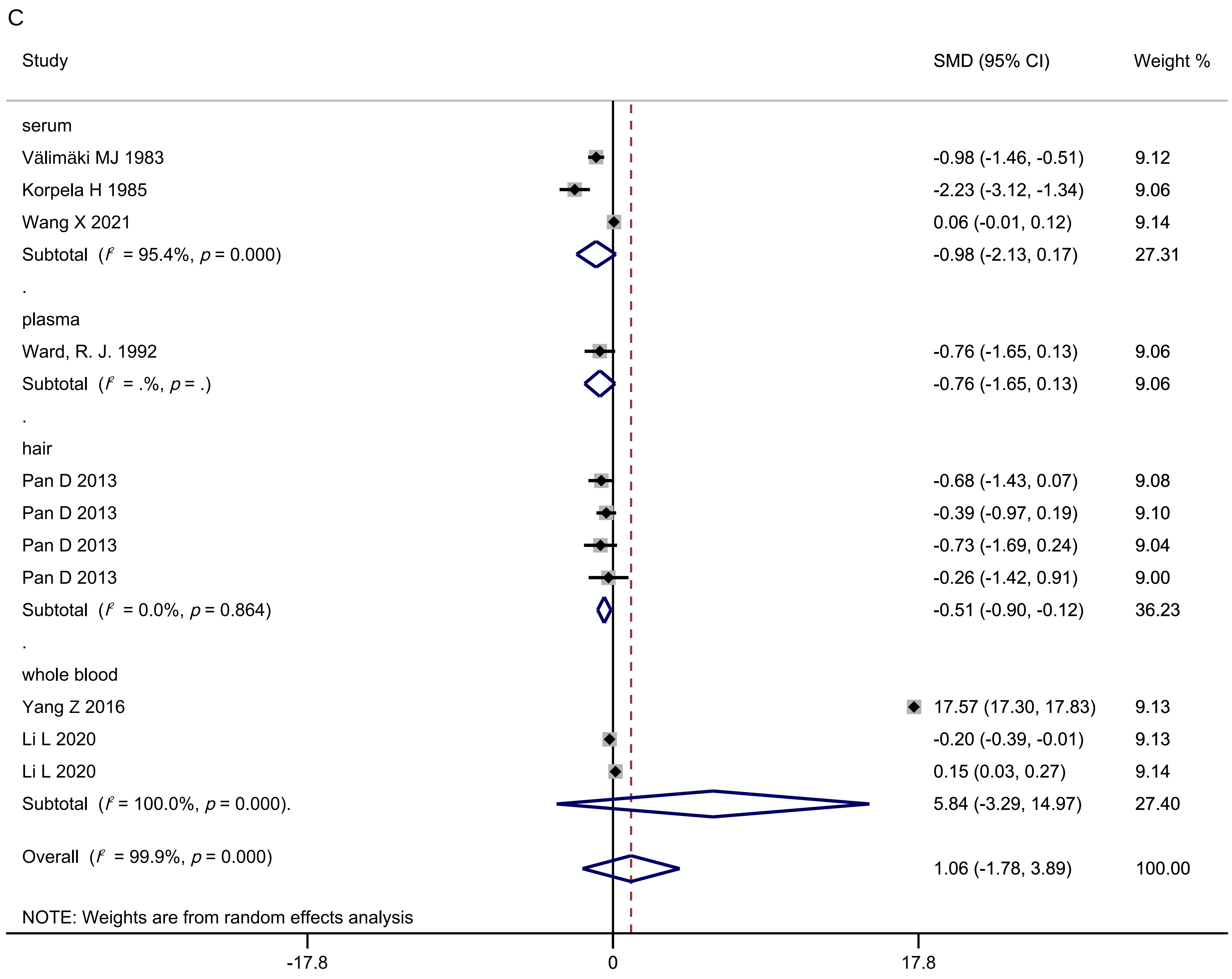
C
Study
SMD (95% CI)
Weight %
serum
Välimäki MJ 1983
-0.98 (-1.46, -0.51)
9.12
Korpela H 1985
-2.23 (-3.12, -1.34)
9.06
Wang X 2021
0.06 (-0.01, 0.12)
9.14
Subtotal (I² = 95.4%, p = 0.000)
-0.98 (-2.13, 0.17)
27.31
plasma
Ward, R. J. 1992
-0.76 (-1.65, 0.13)
9.06
Subtotal (I² = .%, p = .)
-0.76 (-1.65, 0.13)
9.06
hair
Pan D 2013
-0.68 (-1.43, 0.07)
9.08
Pan D 2013
-0.39 (-0.97, 0.19)
9.10
Pan D 2013
-0.73 (-1.69, 0.24)
9.04
Pan D 2013
-0.26 (-1.42, 0.91)
9.00
Subtotal (I² = 0.0%, p = 0.864)
-0.51 (-0.90, -0.12)
36.23
whole blood
Yang Z 2016
17.57 (17.30, 17.83)
9.13
Li L 2020
-0.20 (-0.39, -0.01)
9.13
Li L 2020
0.15 (0.03, 0.27)
9.14
Subtotal (I² = 100.0%, p = 0.000).
5.84 (-3.29, 14.97)
27.40
Overall (I² = 99.9%, p = 0.000)
1.06 (-1.78, 3.89)
100.00
NOTE: Weights are from random effects analysis
-17.8
0
17.8

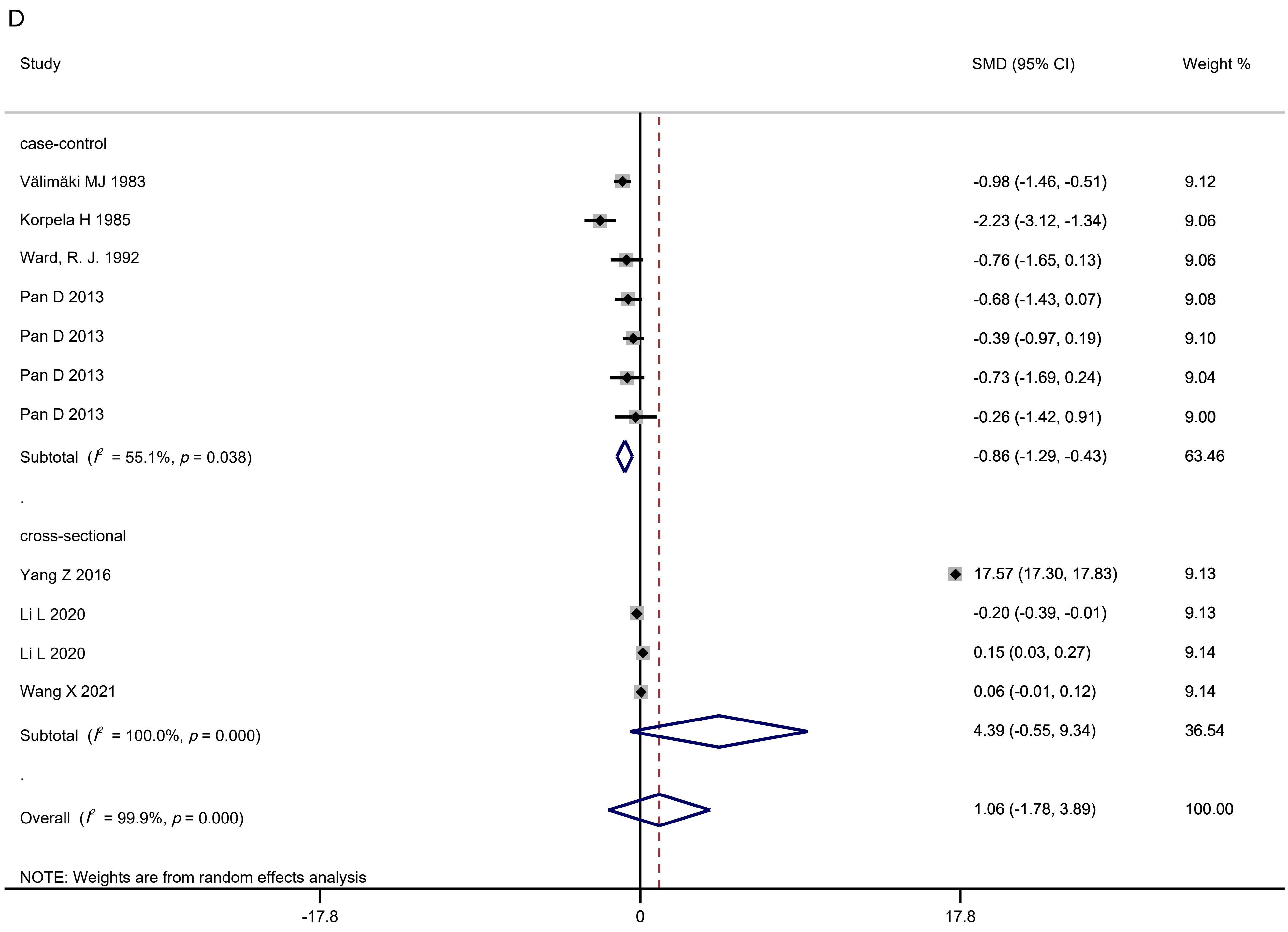
D
Study
SMD (95% CI)
Weight %
case-control
Välimäki MJ 1983
-0.98 (-1.46, -0.51)
9.12
Korpela H 1985
-2.23 (-3.12, -1.34)
9.06
Ward, R. J. 1992
-0.76 (-1.65, 0.13)
9.06
Pan D 2013
-0.68 (-1.43, 0.07)
9.08
Pan D 2013
-0.39 (-0.97, 0.19)
9.10
Pan D 2013
-0.73 (-1.69, 0.24)
9.04
Pan D 2013
-0.26 (-1.42, 0.91)
9.00
Subtotal (I^2 = 55.1%, p = 0.038)
-0.86 (-1.29, -0.43)
63.46
.
cross-sectional
Yang Z 2016
17.57 (17.30, 17.83)
9.13
Li L 2020
-0.20 (-0.39, -0.01)
9.13
Li L 2020
0.15 (0.03, 0.27)
9.14
Wang X 2021
0.06 (-0.01, 0.12)
9.14
Subtotal (I^2 = 100.0%, p = 0.000)
4.39 (-0.55, 9.34)
36.54
.
Overall (I^2 = 99.9%, p = 0.000)
1.06 (-1.78, 3.89)
100.00
NOTE: Weights are from random effects analysis
-17.8
0
17.8

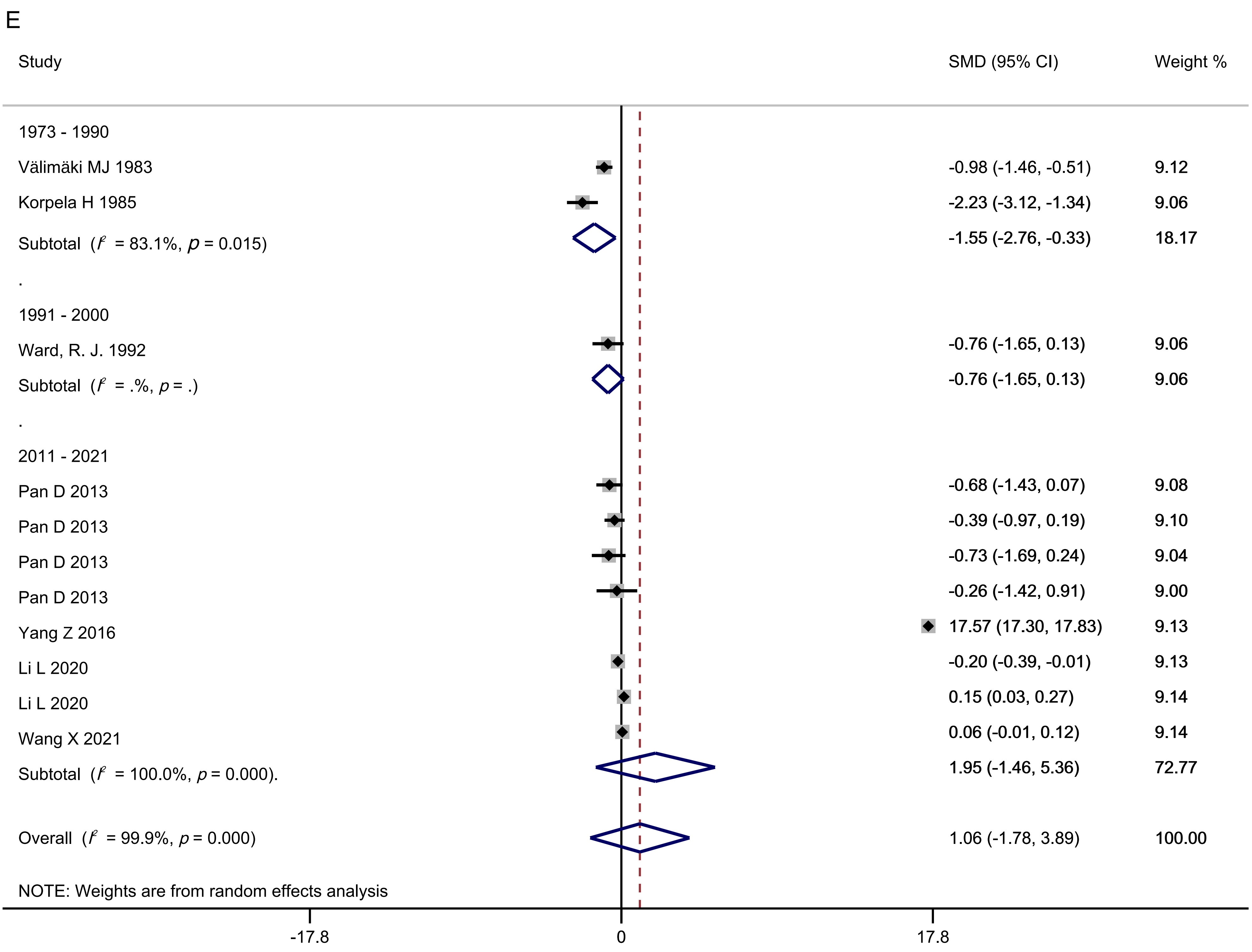
E
Study
SMD (95% CI)
Weight %
1973 - 1990
Välimäki MJ 1983
-0.98 (-1.46, -0.51)
9.12
Korpela H 1985
-2.23 (-3.12, -1.34)
9.06
Subtotal (I^2 = 83.1%, p = 0.015)
-1.55 (-2.76, -0.33)
18.17
.
1991 - 2000
Ward, R. J. 1992
-0.76 (-1.65, 0.13)
9.06
Subtotal (I^2 = .%, p = .)
-0.76 (-1.65, 0.13)
9.06
.
2011 - 2021
Pan D 2013
-0.68 (-1.43, 0.07)
9.08
Pan D 2013
-0.39 (-0.97, 0.19)
9.10
Pan D 2013
-0.73 (-1.69, 0.24)
9.04
Pan D 2013
-0.26 (-1.42, 0.91)
9.00
Yang Z 2016
17.57 (17.30, 17.83)
9.13
Li L 2020
-0.20 (-0.39, -0.01)
9.13
Li L 2020
0.15 (0.03, 0.27)
9.14
Wang X 2021
0.06 (-0.01, 0.12)
9.14
Subtotal (I^2 = 100.0%, p = 0.000).
1.95 (-1.46, 5.36)
72.77
Overall (I^2 = 99.9%, p = 0.000)
1.06 (-1.78, 3.89)
100.00
NOTE: Weights are from random effects analysis
-17.8
0
17.8

F

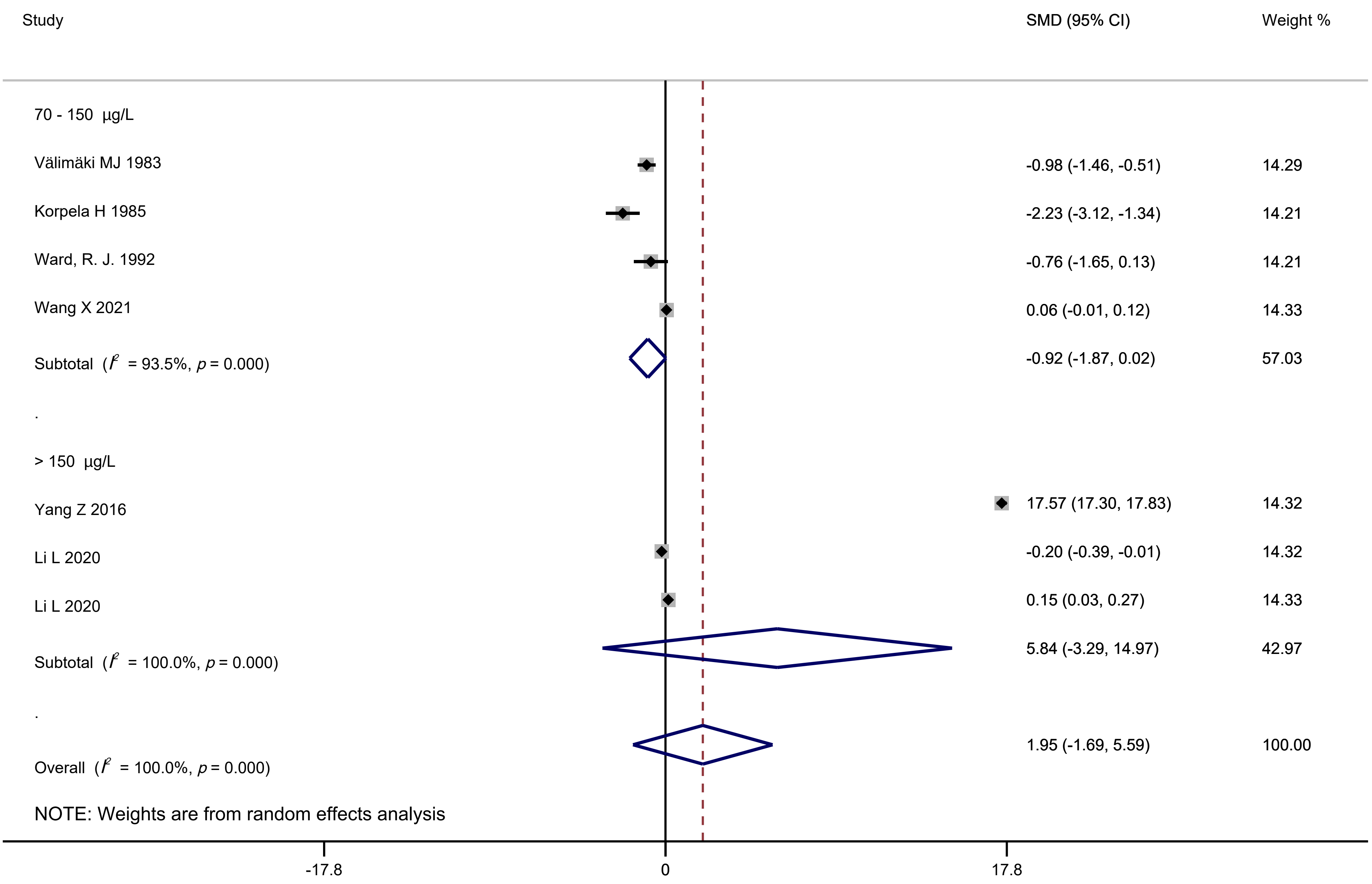

Study
SMD (95% CI)
Weight %
70 - 150 μg/L
Välimäki MJ 1983
-0.98 (-1.46, -0.51)
14.29
Korpela H 1985
-2.23 (-3.12, -1.34)
14.21
Ward, R. J. 1992
-0.76 (-1.65, 0.13)
14.21
Wang X 2021
0.06 (-0.01, 0.12)
14.33
Subtotal (I^2 = 93.5%, p = 0.000)
-0.92 (-1.87, 0.02)
57.03
.
> 150 μg/L
Yang Z 2016
17.57 (17.30, 17.83)
14.32
Li L 2020
-0.20 (-0.39, -0.01)
14.32
Li L 2020
0.15 (0.03, 0.27)
14.33
Subtotal (I^2 = 100.0%, p = 0.000)
5.84 (-3.29, 14.97)
42.97
.
Overall (I^2 = 100.0%, p = 0.000)
1.95 (-1.69, 5.59)
100.00
NOTE: Weights are from random effects analysis
-17.8
0
17.8

Figure S2. Forest plot in the meta-analysis of selenium levels and fatty liver diseases. The dot represents the effect size of each study[16,17,30,32 – 34,77]. The line through the dot represents 95% CI of the effect size. The size of the square represents the weight, Wi. The broken line represents the effect size and the horizontal length of the diamond the 95% CI of the effect size for all studies combined. (A) stratified by disease types;(B) stratified by study regions; (C) stratified by sample sources; (D) stratified by study design; (E) stratified by year of publication; (F) stratified by the reference blood (include whole blood, serum, plasma) selenium levels (70-150 µg/L).

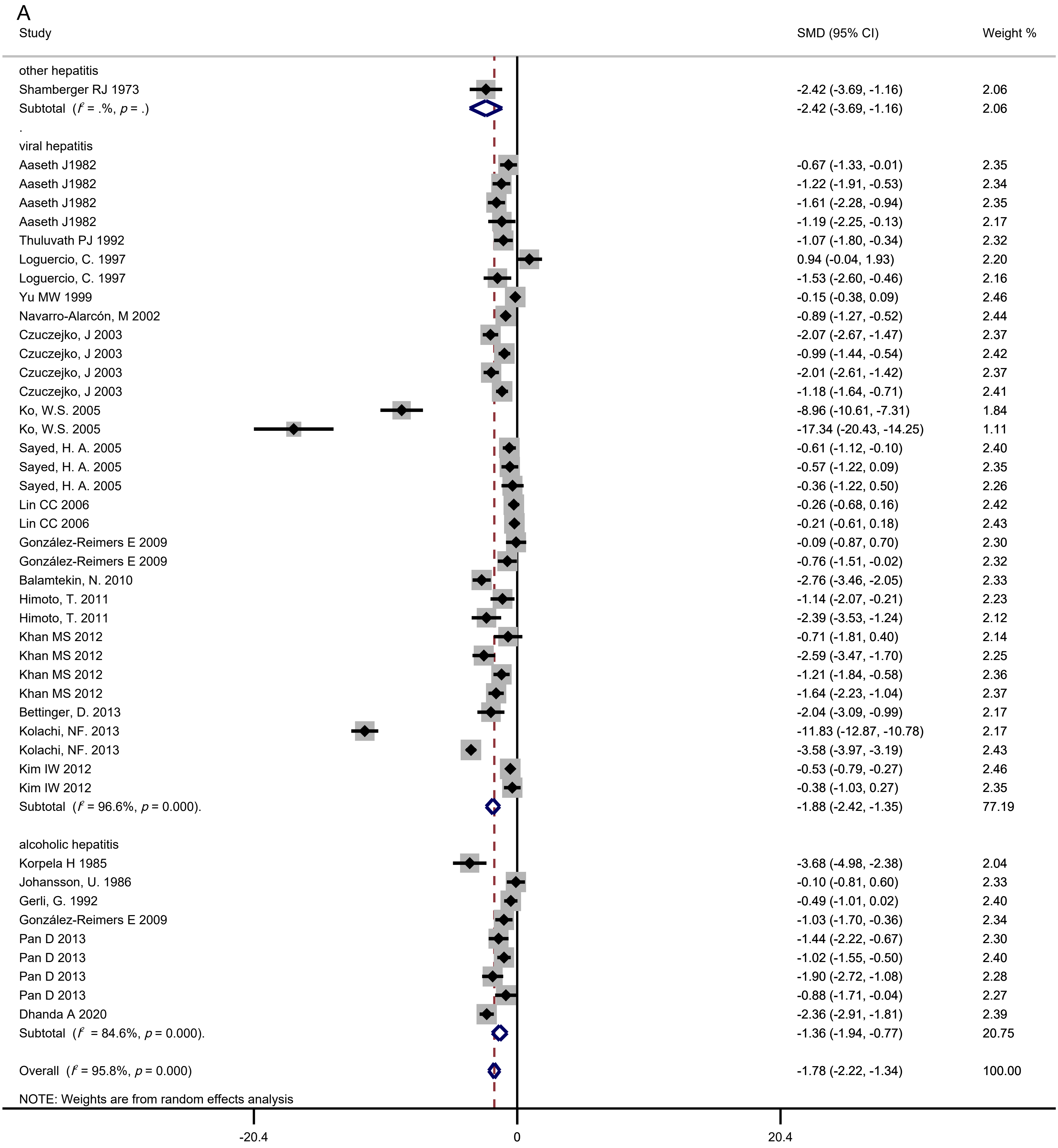
A
Study
SMD (95% CI)
Weight %
other hepatitis
Shamberger RJ 1973 -2.42 (-3.69, -1.16) 2.06
Subtotal (I^2 = .%, p = .) -2.42 (-3.69, -1.16) 2.06
.
viral hepatitis
Aaseth J1982 -0.67 (-1.33, -0.01) 2.35
Aaseth J1982 -1.22 (-1.91, -0.53) 2.34
Aaseth J1982 -1.61 (-2.28, -0.94) 2.35
Aaseth J1982 -1.19 (-2.25, -0.13) 2.17
Thuluvath PJ 1992 -1.07 (-1.80, -0.34) 2.32
Loguercio, C. 1997 0.94 (-0.04, 1.93) 2.20
Loguercio, C. 1997 -1.53 (-2.60, -0.46) 2.16
Yu MW 1999 -0.15 (-0.38, 0.09) 2.46
Navarro-Alarcón, M 2002 -0.89 (-1.27, -0.52) 2.44
Czuczejko, J 2003 -2.07 (-2.67, -1.47) 2.37
Czuczejko, J 2003 -0.99 (-1.44, -0.54) 2.42
Czuczejko, J 2003 -2.01 (-2.61, -1.42) 2.37
Czuczejko, J 2003 -1.18 (-1.64, -0.71) 2.41
Ko, W.S. 2005 -8.96 (-10.61, -7.31) 1.84
Ko, W.S. 2005 -17.34 (-20.43, -14.25) 1.11
Sayed, H. A. 2005 -0.61 (-1.12, -0.10) 2.40
Sayed, H. A. 2005 -0.57 (-1.22, 0.09) 2.35
Sayed, H. A. 2005 -0.36 (-1.22, 0.50) 2.26
Lin CC 2006 -0.26 (-0.68, 0.16) 2.42
Lin CC 2006 -0.21 (-0.61, 0.18) 2.43
González-Reimers E 2009 -0.09 (-0.87, 0.70) 2.30
González-Reimers E 2009 -0.76 (-1.51, -0.02) 2.32
Balamtekin, N. 2010 -2.76 (-3.46, -2.05) 2.33
Himoto, T. 2011 -1.14 (-2.07, -0.21) 2.23
Himoto, T. 2011 -2.39 (-3.53, -1.24) 2.12
Khan MS 2012 -0.71 (-1.81, 0.40) 2.14
Khan MS 2012 -2.59 (-3.47, -1.70) 2.25
Khan MS 2012 -1.21 (-1.84, -0.58) 2.36
Khan MS 2012 -1.64 (-2.23, -1.04) 2.37
Bettinger, D. 2013 -2.04 (-3.09, -0.99) 2.17
Kolachi, NF. 2013 -11.83 (-12.87, -10.78) 2.17
Kolachi, NF. 2013 -3.58 (-3.97, -3.19) 2.43
Kim IW 2012 -0.53 (-0.79, -0.27) 2.46
Kim IW 2012 -0.38 (-1.03, 0.27) 2.35
Subtotal (I^2 = 96.6%, p = 0.000). -1.88 (-2.42, -1.35) 77.19
alcoholic hepatitis
Korpela H 1985 -3.68 (-4.98, -2.38) 2.04
Johansson, U. 1986 -0.10 (-0.81, 0.60) 2.33
Gerli, G. 1992 -0.49 (-1.01, 0.02) 2.40
González-Reimers E 2009 -1.03 (-1.70, -0.36) 2.34
Pan D 2013 -1.44 (-2.22, -0.67) 2.30
Pan D 2013 -1.02 (-1.55, -0.50) 2.40
Pan D 2013 -1.90 (-2.72, -1.08) 2.28
Pan D 2013 -0.88 (-1.71, -0.04) 2.27
Dhanda A 2020 -2.36 (-2.91, -1.81) 2.39
Subtotal (I^2 = 84.6%, p = 0.000). -1.36 (-1.94, -0.77) 20.75
Overall (I^2 = 95.8%, p = 0.000) -1.78 (-2.22, -1.34) 100.00
NOTE: Weights are from random effects analysis
-20.4
0
20.4

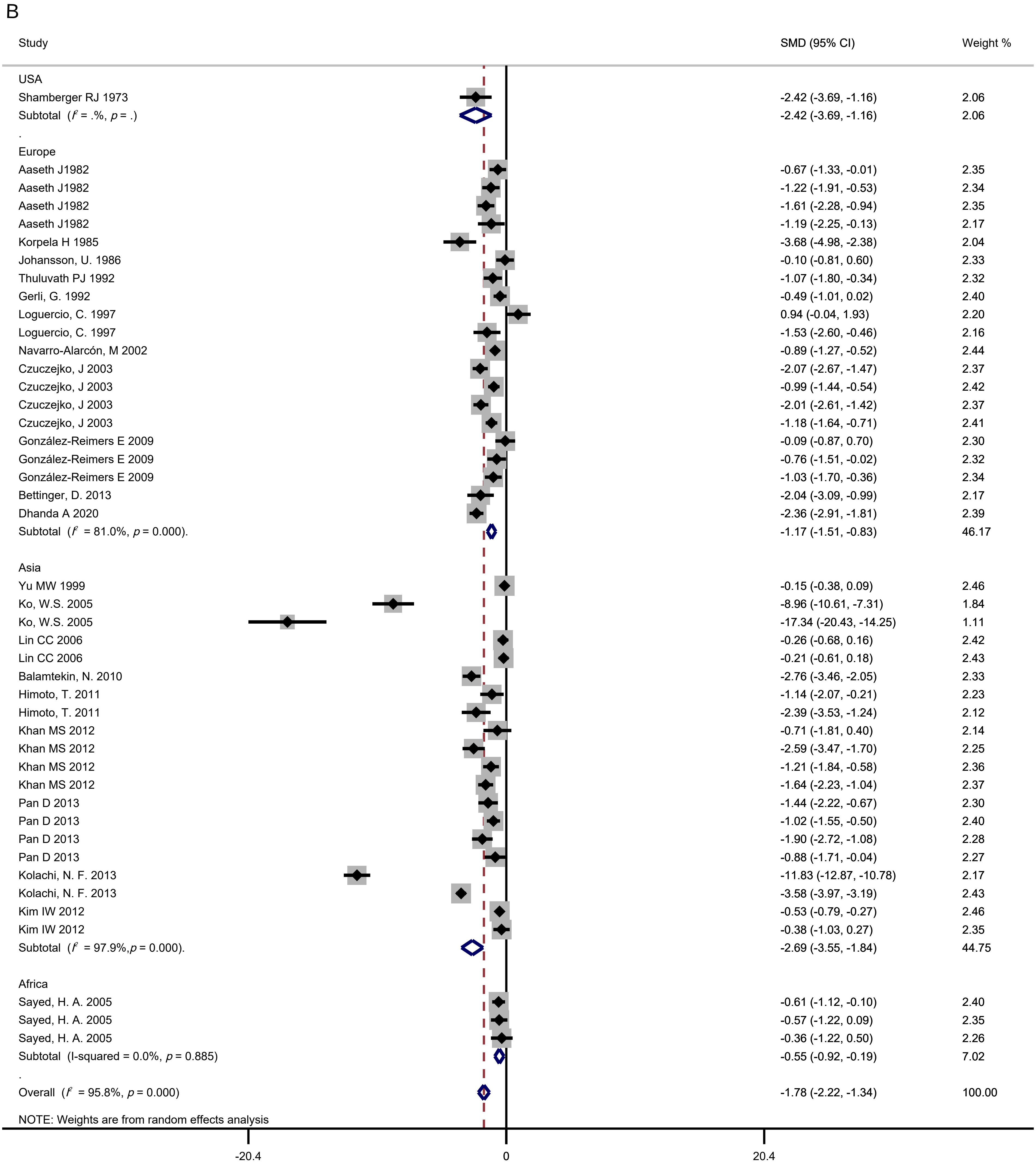
B
Study
SMD (95% CI)
Weight %
USA
Shamberger RJ 1973 -2.42 (-3.69, -1.16) 2.06
Subtotal (I^2 = .%, p = .) -2.42 (-3.69, -1.16) 2.06
.
Europe
Aaseth J1982 -0.67 (-1.33, -0.01) 2.35
Aaseth J1982 -1.22 (-1.91, -0.53) 2.34
Aaseth J1982 -1.61 (-2.28, -0.94) 2.35
Aaseth J1982 -1.19 (-2.25, -0.13) 2.17
Korpela H 1985 -3.68 (-4.98, -2.38) 2.04
Johansson, U. 1986 -0.10 (-0.81, 0.60) 2.33
Thuluvath PJ 1992 -1.07 (-1.80, -0.34) 2.32
Gerli, G. 1992 -0.49 (-1.01, 0.02) 2.40
Loguercio, C. 1997 0.94 (-0.04, 1.93) 2.20
Loguercio, C. 1997 -1.53 (-2.60, -0.46) 2.16
Navarro-Alarcón, M 2002 -0.89 (-1.27, -0.52) 2.44
Czuczejko, J 2003 -2.07 (-2.67, -1.47) 2.37
Czuczejko, J 2003 -0.99 (-1.44, -0.54) 2.42
Czuczejko, J 2003 -2.01 (-2.61, -1.42) 2.37
Czuczejko, J 2003 -1.18 (-1.64, -0.71) 2.41
González-Reimers E 2009 -0.09 (-0.87, 0.70) 2.30
González-Reimers E 2009 -0.76 (-1.51, -0.02) 2.32
González-Reimers E 2009 -1.03 (-1.70, -0.36) 2.34
Bettinger, D. 2013 -2.04 (-3.09, -0.99) 2.17
Dhanda A 2020 -2.36 (-2.91, -1.81) 2.39
Subtotal (I^2 = 81.0%, p = 0.000). -1.17 (-1.51, -0.83) 46.17
Asia
Yu MW 1999 -0.15 (-0.38, 0.09) 2.46
Ko, W.S. 2005 -8.96 (-10.61, -7.31) 1.84
Ko, W.S. 2005 -17.34 (-20.43, -14.25) 1.11
Lin CC 2006 -0.26 (-0.68, 0.16) 2.42
Lin CC 2006 -0.21 (-0.61, 0.18) 2.43
Balamtekin, N. 2010 -2.76 (-3.46, -2.05) 2.33
Himoto, T. 2011 -1.14 (-2.07, -0.21) 2.23
Himoto, T. 2011 -2.39 (-3.53, -1.24) 2.12
Khan MS 2012 -0.71 (-1.81, 0.40) 2.14
Khan MS 2012 -2.59 (-3.47, -1.70) 2.25
Khan MS 2012 -1.21 (-1.84, -0.58) 2.36
Khan MS 2012 -1.64 (-2.23, -1.04) 2.37
Pan D 2013 -1.44 (-2.22, -0.67) 2.30
Pan D 2013 -1.02 (-1.55, -0.50) 2.40
Pan D 2013 -1.90 (-2.72, -1.08) 2.28
Pan D 2013 -0.88 (-1.71, -0.04) 2.27
Kolachi, N. F. 2013 -11.83 (-12.87, -10.78) 2.17
Kolachi, N. F. 2013 -3.58 (-3.97, -3.19) 2.43
Kim IW 2012 -0.53 (-0.79, -0.27) 2.46
Kim IW 2012 -0.38 (-1.03, 0.27) 2.35
Subtotal (I^2 = 97.9%, p = 0.000). -2.69 (-3.55, -1.84) 44.75
Africa
Sayed, H. A. 2005 -0.61 (-1.12, -0.10) 2.40
Sayed, H. A. 2005 -0.57 (-1.22, 0.09) 2.35
Sayed, H. A. 2005 -0.36 (-1.22, 0.50) 2.26
Subtotal (I-squared = 0.0%, p = 0.885) -0.55 (-0.92, -0.19) 7.02
.
Overall (I^2 = 95.8%, p = 0.000) -1.78 (-2.22, -1.34) 100.00
NOTE: Weights are from random effects analysis
-20.4
0
20.4

C

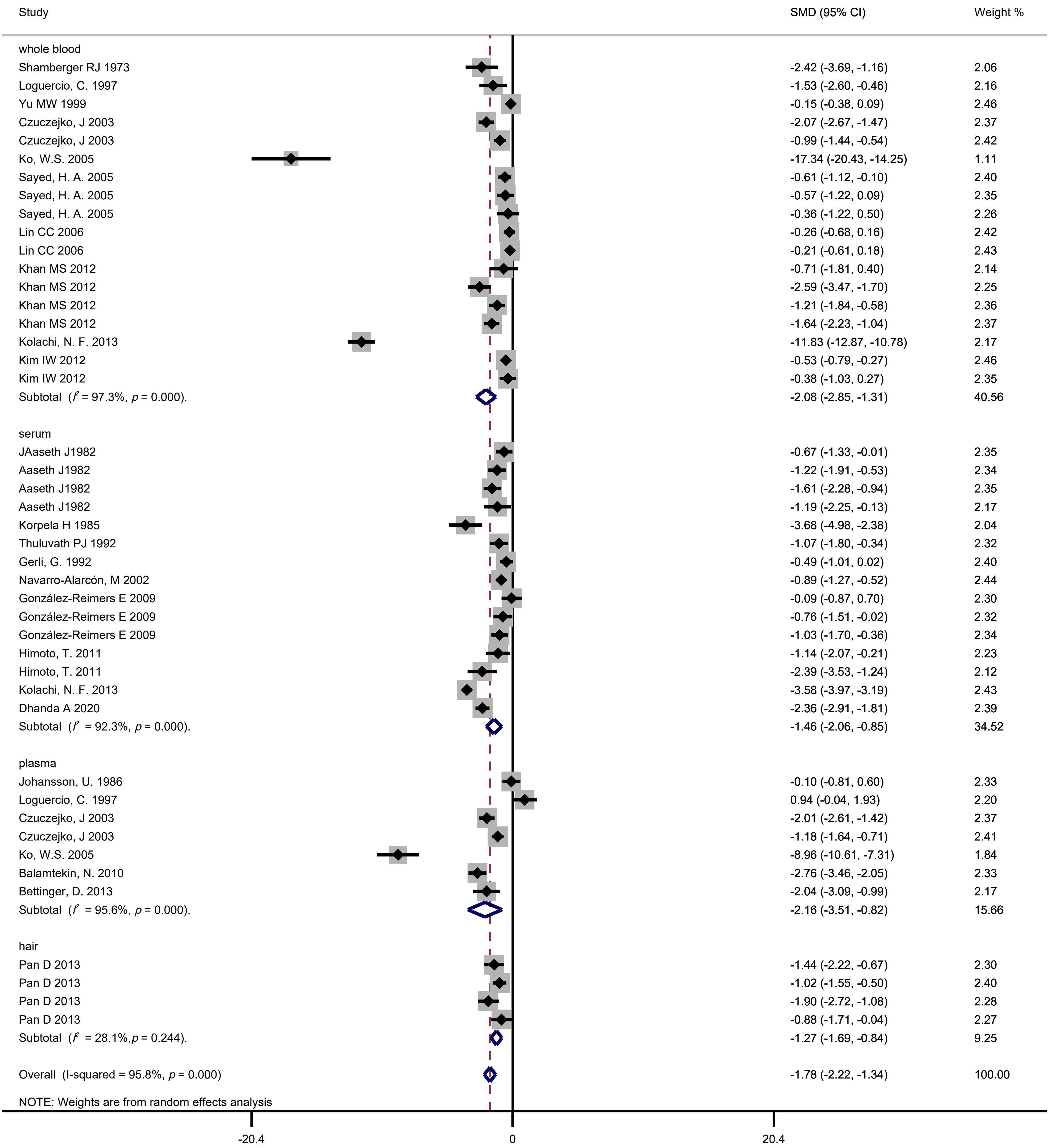

Study
SMD (95% CI)
Weight %
whole blood
Shamberger RJ 1973 -2.42 (-3.69, -1.16) 2.06
Loguercio, C. 1997 -1.53 (-2.60, -0.46) 2.16
Yu MW 1999 -0.15 (-0.38, 0.09) 2.46
Czuczejko, J 2003 -2.07 (-2.67, -1.47) 2.37
Czuczejko, J 2003 -0.99 (-1.44, -0.54) 2.42
Ko, W.S. 2005 -17.34 (-20.43, -14.25) 1.11
Sayed, H. A. 2005 -0.61 (-1.12, -0.10) 2.40
Sayed, H. A. 2005 -0.57 (-1.22, 0.09) 2.35
Sayed, H. A. 2005 -0.36 (-1.22, 0.50) 2.26
Lin CC 2006 -0.26 (-0.68, 0.16) 2.42
Lin CC 2006 -0.21 (-0.61, 0.18) 2.43
Khan MS 2012 -0.71 (-1.81, 0.40) 2.14
Khan MS 2012 -2.59 (-3.47, -1.70) 2.25
Khan MS 2012 -1.21 (-1.84, -0.58) 2.36
Khan MS 2012 -1.64 (-2.23, -1.04) 2.37
Kolachi, N. F. 2013 -11.83 (-12.87, -10.78) 2.17
Kim IW 2012 -0.53 (-0.79, -0.27) 2.46
Kim IW 2012 -0.38 (-1.03, 0.27) 2.35
Subtotal (I^2 = 97.3%, p = 0.000). -2.08 (-2.85, -1.31) 40.56
serum
JAaseth J1982 -0.67 (-1.33, -0.01) 2.35
Aaseth J1982 -1.22 (-1.91, -0.53) 2.34
Aaseth J1982 -1.61 (-2.28, -0.94) 2.35
Aaseth J1982 -1.19 (-2.25, -0.13) 2.17
Korpela H 1985 -3.68 (-4.98, -2.38) 2.04
Thuluvath PJ 1992 -1.07 (-1.80, -0.34) 2.32
Gerli, G. 1992 -0.49 (-1.01, 0.02) 2.40
Navarro-Alarcón, M 2002 -0.89 (-1.27, -0.52) 2.44
González-Reimers E 2009 -0.09 (-0.87, 0.70) 2.30
González-Reimers E 2009 -0.76 (-1.51, -0.02) 2.32
González-Reimers E 2009 -1.03 (-1.70, -0.36) 2.34
Himoto, T. 2011 -1.14 (-2.07, -0.21) 2.23
Himoto, T. 2011 -2.39 (-3.53, -1.24) 2.12
Kolachi, N. F. 2013 -3.58 (-3.97, -3.19) 2.43
Dhanda A 2020 -2.36 (-2.91, -1.81) 2.39
Subtotal (I^2 = 92.3%, p = 0.000). -1.46 (-2.06, -0.85) 34.52
plasma
Johansson, U. 1986 -0.10 (-0.81, 0.60) 2.33
Loguercio, C. 1997 0.94 (-0.04, 1.93) 2.20
Czuczejko, J 2003 -2.01 (-2.61, -1.42) 2.37
Czuczejko, J 2003 -1.18 (-1.64, -0.71) 2.41
Ko, W.S. 2005 -8.96 (-10.61, -7.31) 1.84
Balamtekin, N. 2010 -2.76 (-3.46, -2.05) 2.33
Bettinger, D. 2013 -2.04 (-3.09, -0.99) 2.17
Subtotal (I^2 = 95.6%, p = 0.000). -2.16 (-3.51, -0.82) 15.66
hair
Pan D 2013 -1.44 (-2.22, -0.67) 2.30
Pan D 2013 -1.02 (-1.55, -0.50) 2.40
Pan D 2013 -1.90 (-2.72, -1.08) 2.28
Pan D 2013 -0.88 (-1.71, -0.04) 2.27
Subtotal (I^2 = 28.1%,p = 0.244). -1.27 (-1.69, -0.84) 9.25
Overall (I-squared = 95.8%, p = 0.000) -1.78 (-2.22, -1.34) 100.00
NOTE: Weights are from random effects analysis
-20.4
0
20.4

D

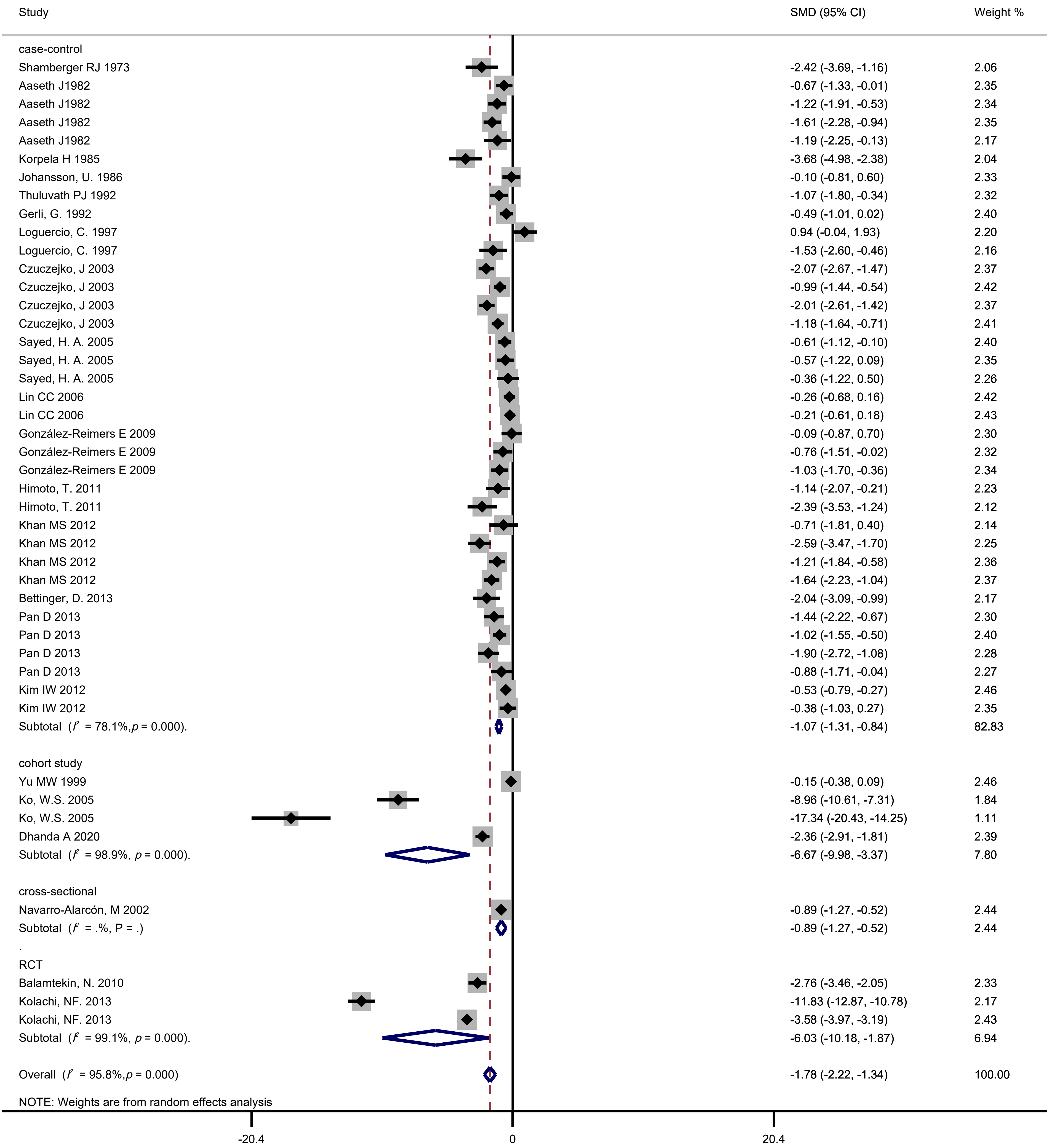
Study
SMD (95% CI)
Weight %
case-control
Shamberger RJ 1973 -2.42 (-3.69, -1.16) 2.06
Aaseth J1982 -0.67 (-1.33, -0.01) 2.35
Aaseth J1982 -1.22 (-1.91, -0.53) 2.34
Aaseth J1982 -1.61 (-2.28, -0.94) 2.35
Aaseth J1982 -1.19 (-2.25, -0.13) 2.17
Korpela H 1985 -3.68 (-4.98, -2.38) 2.04
Johansson, U. 1986 -0.10 (-0.81, 0.60) 2.33
Thuluvath PJ 1992 -1.07 (-1.80, -0.34) 2.32
Gerli, G. 1992 -0.49 (-1.01, 0.02) 2.40
Loguercio, C. 1997 0.94 (-0.04, 1.93) 2.20
Loguercio, C. 1997 -1.53 (-2.60, -0.46) 2.16
Czuczejko, J 2003 -2.07 (-2.67, -1.47) 2.37
Czuczejko, J 2003 -0.99 (-1.44, -0.54) 2.42
Czuczejko, J 2003 -2.01 (-2.61, -1.42) 2.37
Czuczejko, J 2003 -1.18 (-1.64, -0.71) 2.41
Sayed, H. A. 2005 -0.61 (-1.12, -0.10) 2.40
Sayed, H. A. 2005 -0.57 (-1.22, 0.09) 2.35
Sayed, H. A. 2005 -0.36 (-1.22, 0.50) 2.26
Lin CC 2006 -0.26 (-0.68, 0.16) 2.42
Lin CC 2006 -0.21 (-0.61, 0.18) 2.43
González-Reimers E 2009 -0.09 (-0.87, 0.70) 2.30
González-Reimers E 2009 -0.76 (-1.51, -0.02) 2.32
González-Reimers E 2009 -1.03 (-1.70, -0.36) 2.34
Himoto, T. 2011 -1.14 (-2.07, -0.21) 2.23
Himoto, T. 2011 -2.39 (-3.53, -1.24) 2.12
Khan MS 2012 -0.71 (-1.81, 0.40) 2.14
Khan MS 2012 -2.59 (-3.47, -1.70) 2.25
Khan MS 2012 -1.21 (-1.84, -0.58) 2.36
Khan MS 2012 -1.64 (-2.23, -1.04) 2.37
Bettinger, D. 2013 -2.04 (-3.09, -0.99) 2.17
Pan D 2013 -1.44 (-2.22, -0.67) 2.30
Pan D 2013 -1.02 (-1.55, -0.50) 2.40
Pan D 2013 -1.90 (-2.72, -1.08) 2.28
Pan D 2013 -0.88 (-1.71, -0.04) 2.27
Kim IW 2012 -0.53 (-0.79, -0.27) 2.46
Kim IW 2012 -0.38 (-1.03, 0.27) 2.35
Subtotal (I^2 = 78.1%,p = 0.000). -1.07 (-1.31, -0.84) 82.83
cohort study
Yu MW 1999 -0.15 (-0.38, 0.09) 2.46
Ko, W.S. 2005 -8.96 (-10.61, -7.31) 1.84
Ko, W.S. 2005 -17.34 (-20.43, -14.25) 1.11
Dhanda A 2020 -2.36 (-2.91, -1.81) 2.39
Subtotal (I^2 = 98.9%, p = 0.000). -6.67 (-9.98, -3.37) 7.80
cross-sectional
Navarro-Alarcón, M 2002 -0.89 (-1.27, -0.52) 2.44
Subtotal (I^2 = .%, P = .) -0.89 (-1.27, -0.52) 2.44
.
RCT
Balamtekin, N. 2010 -2.76 (-3.46, -2.05) 2.33
Kolachi, NF. 2013 -11.83 (-12.87, -10.78) 2.17
Kolachi, NF. 2013 -3.58 (-3.97, -3.19) 2.43
Subtotal (I^2 = 99.1%, p = 0.000). -6.03 (-10.18, -1.87) 6.94
Overall (I^2 = 95.8%,p = 0.000) -1.78 (-2.22, -1.34) 100.00
NOTE: Weights are from random effects analysis
-20.4
0
20.4

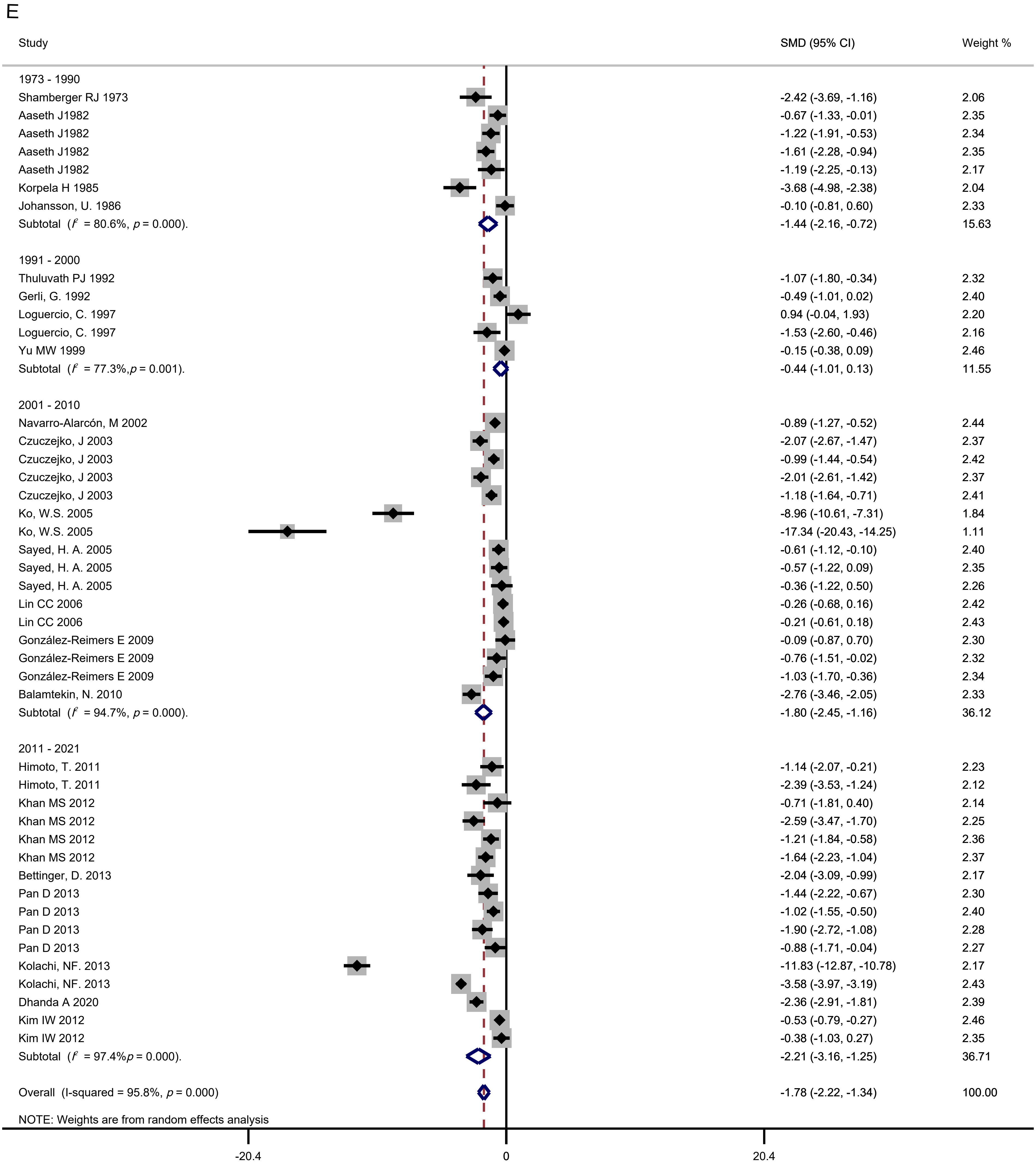
E
Study
SMD (95% CI)
Weight %
1973 - 1990
Shamberger RJ 1973 -2.42 (-3.69, -1.16) 2.06
Aaseth J1982 -0.67 (-1.33, -0.01) 2.35
Aaseth J1982 -1.22 (-1.91, -0.53) 2.34
Aaseth J1982 -1.61 (-2.28, -0.94) 2.35
Aaseth J1982 -1.19 (-2.25, -0.13) 2.17
Korpela H 1985 -3.68 (-4.98, -2.38) 2.04
Johansson, U. 1986 -0.10 (-0.81, 0.60) 2.33
Subtotal (I^2 = 80.6%, p = 0.000). -1.44 (-2.16, -0.72) 15.63
1991 - 2000
Thuluvath PJ 1992 -1.07 (-1.80, -0.34) 2.32
Gerli, G. 1992 -0.49 (-1.01, 0.02) 2.40
Loguercio, C. 1997 0.94 (-0.04, 1.93) 2.20
Loguercio, C. 1997 -1.53 (-2.60, -0.46) 2.16
Yu MW 1999 -0.15 (-0.38, 0.09) 2.46
Subtotal (I^2 = 77.3%,p = 0.001). -0.44 (-1.01, 0.13) 11.55
2001 - 2010
Navarro-Alarcón, M 2002 -0.89 (-1.27, -0.52) 2.44
Czuczejko, J 2003 -2.07 (-2.67, -1.47) 2.37
Czuczejko, J 2003 -0.99 (-1.44, -0.54) 2.42
Czuczejko, J 2003 -2.01 (-2.61, -1.42) 2.37
Czuczejko, J 2003 -1.18 (-1.64, -0.71) 2.41
Ko, W.S. 2005 -8.96 (-10.61, -7.31) 1.84
Ko, W.S. 2005 -17.34 (-20.43, -14.25) 1.11
Sayed, H. A. 2005 -0.61 (-1.12, -0.10) 2.40
Sayed, H. A. 2005 -0.57 (-1.22, 0.09) 2.35
Sayed, H. A. 2005 -0.36 (-1.22, 0.50) 2.26
Lin CC 2006 -0.26 (-0.68, 0.16) 2.42
Lin CC 2006 -0.21 (-0.61, 0.18) 2.43
González-Reimers E 2009 -0.09 (-0.87, 0.70) 2.30
González-Reimers E 2009 -0.76 (-1.51, -0.02) 2.32
González-Reimers E 2009 -1.03 (-1.70, -0.36) 2.34
Balamtekin, N. 2010 -2.76 (-3.46, -2.05) 2.33
Subtotal (I^2 = 94.7%, p = 0.000). -1.80 (-2.45, -1.16) 36.12
2011 - 2021
Himoto, T. 2011 -1.14 (-2.07, -0.21) 2.23
Himoto, T. 2011 -2.39 (-3.53, -1.24) 2.12
Khan MS 2012 -0.71 (-1.81, 0.40) 2.14
Khan MS 2012 -2.59 (-3.47, -1.70) 2.25
Khan MS 2012 -1.21 (-1.84, -0.58) 2.36
Khan MS 2012 -1.64 (-2.23, -1.04) 2.37
Bettinger, D. 2013 -2.04 (-3.09, -0.99) 2.17
Pan D 2013 -1.44 (-2.22, -0.67) 2.30
Pan D 2013 -1.02 (-1.55, -0.50) 2.40
Pan D 2013 -1.90 (-2.72, -1.08) 2.28
Pan D 2013 -0.88 (-1.71, -0.04) 2.27
Kolachi, NF. 2013 -11.83 (-12.87, -10.78) 2.17
Kolachi, NF. 2013 -3.58 (-3.97, -3.19) 2.43
Dhanda A 2020 -2.36 (-2.91, -1.81) 2.39
Kim IW 2012 -0.53 (-0.79, -0.27) 2.46
Kim IW 2012 -0.38 (-1.03, 0.27) 2.35
Subtotal (I^2 = 97.4%p = 0.000). -2.21 (-3.16, -1.25) 36.71
Overall (I-squared = 95.8%, p = 0.000) -1.78 (-2.22, -1.34) 100.00
NOTE: Weights are from random effects analysis
-20.4
0
20.4

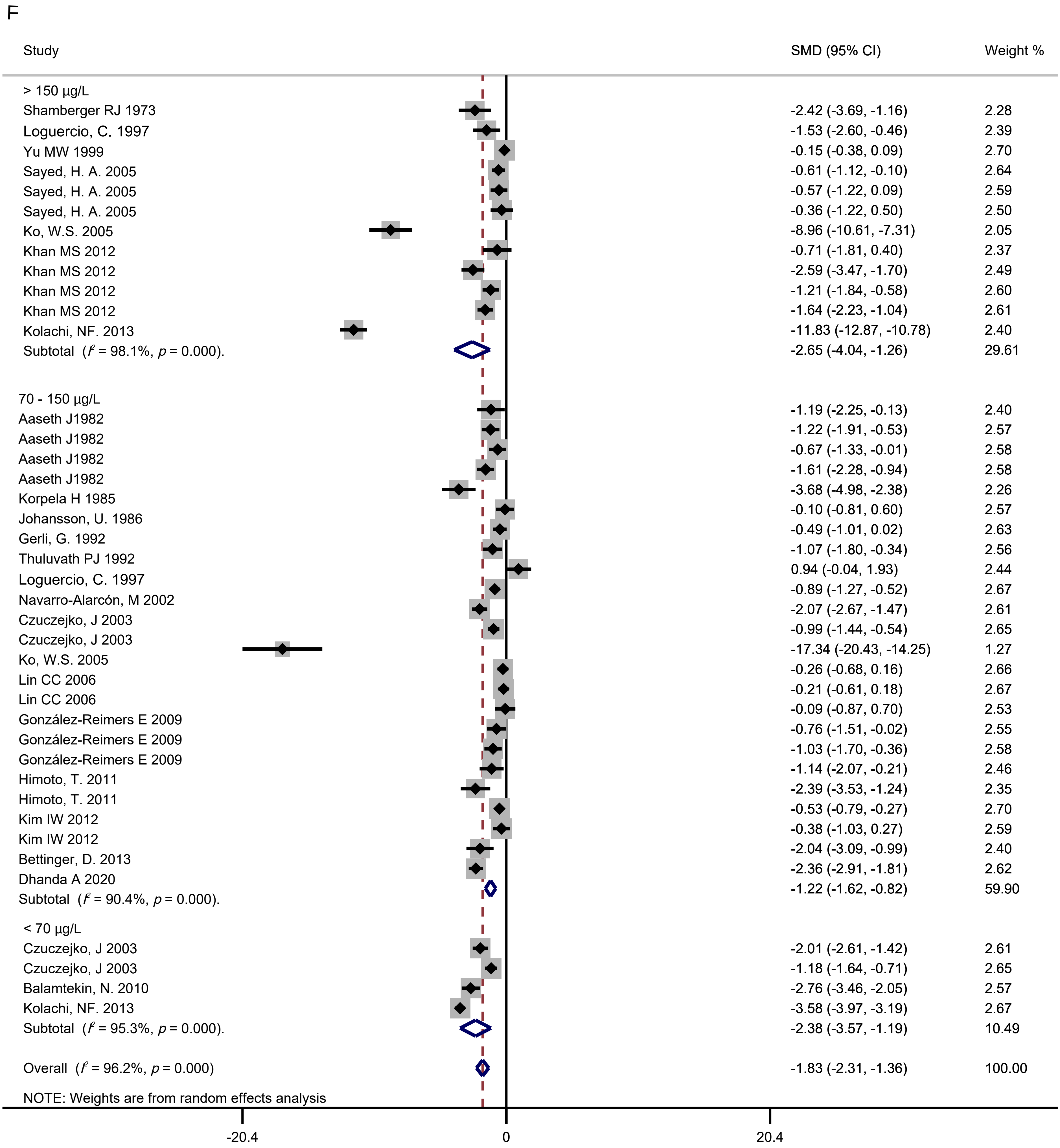
F
Study
SMD (95% CI)
Weight %
> 150 µg/L
Shamberger RJ 1973
Loguercio, C. 1997
Yu MW 1999
Sayed, H. A. 2005
Sayed, H. A. 2005
Sayed, H. A. 2005
Ko, W.S. 2005
Khan MS 2012
Khan MS 2012
Khan MS 2012
Khan MS 2012
Kolachi, NF. 2013
Subtotal (I^2 = 98.1%, p = 0.000).
70 - 150 µg/L
Aaseth J1982
Aaseth J1982
Aaseth J1982
Aaseth J1982
Korpela H 1985
Johansson, U. 1986
Gerli, G. 1992
Thuluvath PJ 1992
Loguercio, C. 1997
Navarro-Alarcón, M 2002
Czuczejko, J 2003
Czuczejko, J 2003
Ko, W.S. 2005
Lin CC 2006
Lin CC 2006
González-Reimers E 2009
González-Reimers E 2009
González-Reimers E 2009
Himoto, T. 2011
Himoto, T. 2011
Kim IW 2012
Kim IW 2012
Bettinger, D. 2013
Dhanda A 2020
Subtotal (I^2 = 90.4%, p = 0.000).
< 70 µg/L
Czuczejko, J 2003
Czuczejko, J 2003
Balamtekin, N. 2010
Kolachi, NF. 2013
Subtotal (I^2 = 95.3%, p = 0.000).
Overall (I^2 = 96.2%, p = 0.000)
NOTE: Weights are from random effects analysis
-2.42 (-3.69, -1.16) 2.28
-1.53 (-2.60, -0.46) 2.39
-0.15 (-0.38, 0.09) 2.70
-0.61 (-1.12, -0.10) 2.64
-0.57 (-1.22, 0.09) 2.59
-0.36 (-1.22, 0.50) 2.50
-8.96 (-10.61, -7.31) 2.05
-0.71 (-1.81, 0.40) 2.37
-2.59 (-3.47, -1.70) 2.49
-1.21 (-1.84, -0.58) 2.60
-1.64 (-2.23, -1.04) 2.61
-11.83 (-12.87, -10.78) 2.40
-2.65 (-4.04, -1.26) 29.61
-1.19 (-2.25, -0.13) 2.40
-1.22 (-1.91, -0.53) 2.57
-0.67 (-1.33, -0.01) 2.58
-1.61 (-2.28, -0.94) 2.58
-3.68 (-4.98, -2.38) 2.26
-0.10 (-0.81, 0.60) 2.57
-0.49 (-1.01, 0.02) 2.63
-1.07 (-1.80, -0.34) 2.56
0.94 (-0.04, 1.93) 2.44
-0.89 (-1.27, -0.52) 2.67
-2.07 (-2.67, -1.47) 2.61
-0.99 (-1.44, -0.54) 2.65
-17.34 (-20.43, -14.25) 1.27
-0.26 (-0.68, 0.16) 2.66
-0.21 (-0.61, 0.18) 2.67
-0.09 (-0.87, 0.70) 2.53
-0.76 (-1.51, -0.02) 2.55
-1.03 (-1.70, -0.36) 2.58
-1.14 (-2.07, -0.21) 2.46
-2.39 (-3.53, -1.24) 2.35
-0.53 (-0.79, -0.27) 2.70
-0.38 (-1.03, 0.27) 2.59
-2.04 (-3.09, -0.99) 2.40
-2.36 (-2.91, -1.81) 2.62
-1.22 (-1.62, -0.82) 59.90
-2.01 (-2.61, -1.42) 2.61
-1.18 (-1.64, -0.71) 2.65
-2.76 (-3.46, -2.05) 2.57
-3.58 (-3.97, -3.19) 2.67
-2.38 (-3.57, -1.19) 10.49
-1.83 (-2.31, -1.36) 100.00
-20.4
0
20.4

Figure S3. Forest plot in the meta-analysis of selenium levels and hepatitis. The dot represents the effect size of each study. The line through the dot represents 95% CI of the effect size[13,19,34-39,40-53]. The size of the square represents the weight, Wi. The broken line represents the effect size and the horizontal length of the diamond the 95% CI of the effect size for all studies combined. (A) stratified by disease types;(B) stratified by study regions; (C) stratified by sample sources; (D) stratified by study design; (E) stratified by year of publication; (F) stratified by the reference blood (include whole blood, serum, plasma) selenium levels (70-150 μg/L).

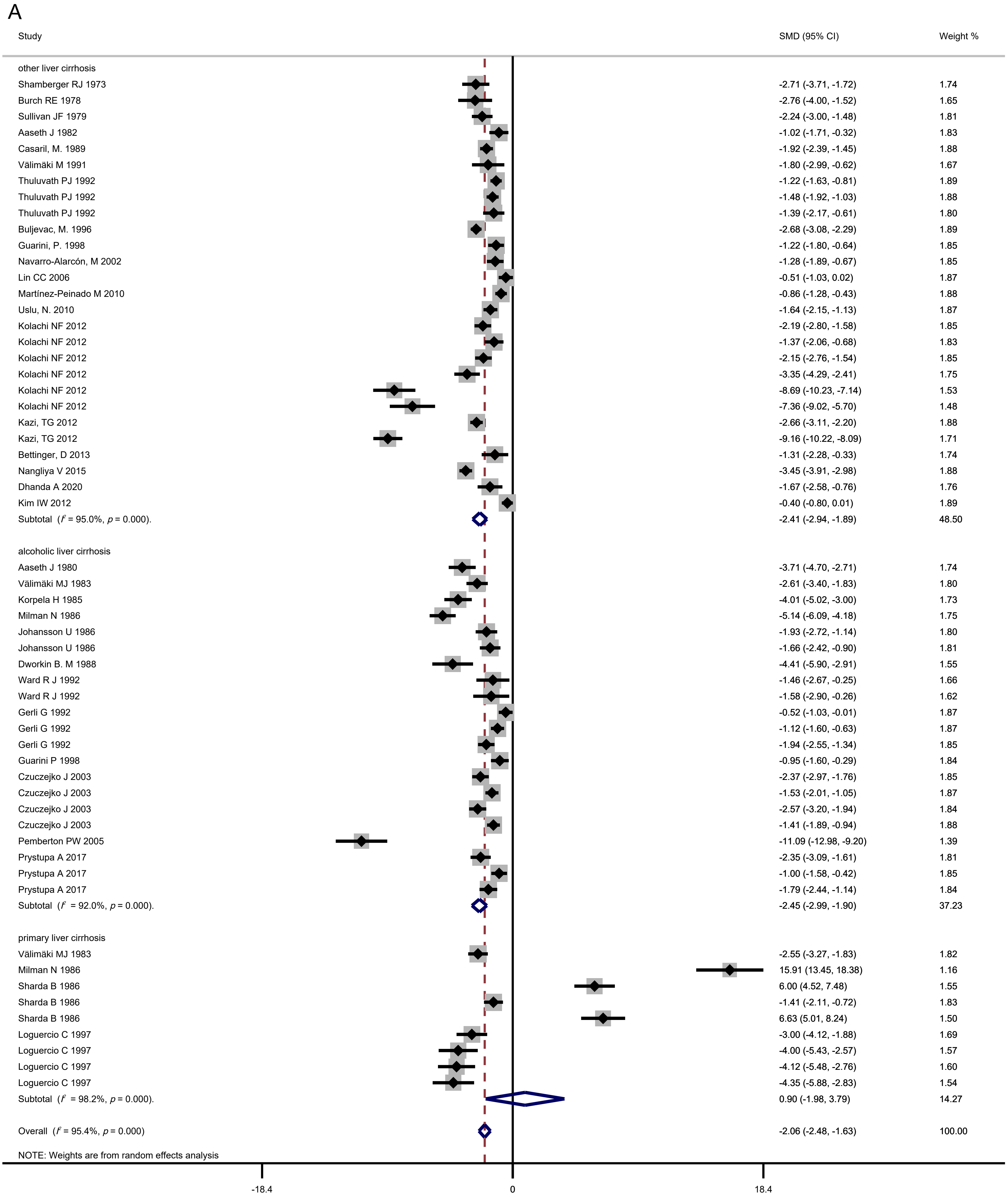
A
Study
SMD (95% CI)
Weight %
other liver cirrhosis
Shamberger RJ 1973 -2.71 (-3.71, -1.72) 1.74
Burch RE 1978 -2.76 (-4.00, -1.52) 1.65
Sullivan JF 1979 -2.24 (-3.00, -1.48) 1.81
Aaseth J 1982 -1.02 (-1.71, -0.32) 1.83
Casaril, M. 1989 -1.92 (-2.39, -1.45) 1.88
Välimäki M 1991 -1.80 (-2.99, -0.62) 1.67
Thuluvath PJ 1992 -1.22 (-1.63, -0.81) 1.89
Thuluvath PJ 1992 -1.48 (-1.92, -1.03) 1.88
Thuluvath PJ 1992 -1.39 (-2.17, -0.61) 1.80
Buljevac, M. 1996 -2.68 (-3.08, -2.29) 1.89
Guarini, P. 1998 -1.22 (-1.80, -0.64) 1.85
Navarro-Alarcón, M 2002 -1.28 (-1.89, -0.67) 1.85
Lin CC 2006 -0.51 (-1.03, 0.02) 1.87
Martínez-Peinado M 2010 -0.86 (-1.28, -0.43) 1.88
Uslu, N. 2010 -1.64 (-2.15, -1.13) 1.87
Kolachi NF 2012 -2.19 (-2.80, -1.58) 1.85
Kolachi NF 2012 -1.37 (-2.06, -0.68) 1.83
Kolachi NF 2012 -2.15 (-2.76, -1.54) 1.85
Kolachi NF 2012 -3.35 (-4.29, -2.41) 1.75
Kolachi NF 2012 -8.69 (-10.23, -7.14) 1.53
Kolachi NF 2012 -7.36 (-9.02, -5.70) 1.48
Kazi, TG 2012 -2.66 (-3.11, -2.20) 1.88
Kazi, TG 2012 -9.16 (-10.22, -8.09) 1.71
Bettinger, D 2013 -1.31 (-2.28, -0.33) 1.74
Nangliya V 2015 -3.45 (-3.91, -2.98) 1.88
Dhanda A 2020 -1.67 (-2.58, -0.76) 1.76
Kim IW 2012 -0.40 (-0.80, 0.01) 1.89
Subtotal (I^2 = 95.0%, p = 0.000). -2.41 (-2.94, -1.89) 48.50
alcoholic liver cirrhosis
Aaseth J 1980 -3.71 (-4.70, -2.71) 1.74
Välimäki MJ 1983 -2.61 (-3.40, -1.83) 1.80
Korpela H 1985 -4.01 (-5.02, -3.00) 1.73
Milman N 1986 -5.14 (-6.09, -4.18) 1.75
Johansson U 1986 -1.93 (-2.72, -1.14) 1.80
Johansson U 1986 -1.66 (-2.42, -0.90) 1.81
Dworkin B. M 1988 -4.41 (-5.90, -2.91) 1.55
Ward R J 1992 -1.46 (-2.67, -0.25) 1.66
Ward R J 1992 -1.58 (-2.90, -0.26) 1.62
Gerli G 1992 -0.52 (-1.03, -0.01) 1.87
Gerli G 1992 -1.12 (-1.60, -0.63) 1.87
Gerli G 1992 -1.94 (-2.55, -1.34) 1.85
Guarini P 1998 -0.95 (-1.60, -0.29) 1.84
Czuczejko J 2003 -2.37 (-2.97, -1.76) 1.85
Czuczejko J 2003 -1.53 (-2.01, -1.05) 1.87
Czuczejko J 2003 -2.57 (-3.20, -1.94) 1.84
Czuczejko J 2003 -1.41 (-1.89, -0.94) 1.88
Pemberton PW 2005 -11.09 (-12.98, -9.20) 1.39
Prystupa A 2017 -2.35 (-3.09, -1.61) 1.81
Prystupa A 2017 -1.00 (-1.58, -0.42) 1.85
Prystupa A 2017 -1.79 (-2.44, -1.14) 1.84
Subtotal (I^2 = 92.0%, p = 0.000). -2.45 (-2.99, -1.90) 37.23
primary liver cirrhosis
Välimäki MJ 1983 -2.55 (-3.27, -1.83) 1.82
Milman N 1986 15.91 (13.45, 18.38) 1.16
Sharda B 1986 6.00 (4.52, 7.48) 1.55
Sharda B 1986 -1.41 (-2.11, -0.72) 1.83
Sharda B 1986 6.63 (5.01, 8.24) 1.50
Loguercio C 1997 -3.00 (-4.12, -1.88) 1.69
Loguercio C 1997 -4.00 (-5.43, -2.57) 1.57
Loguercio C 1997 -4.12 (-5.48, -2.76) 1.60
Loguercio C 1997 -4.35 (-5.88, -2.83) 1.54
Subtotal (I^2 = 98.2%, p = 0.000). 0.90 (-1.98, 3.79) 14.27
Overall (I^2 = 95.4%, p = 0.000) -2.06 (-2.48, -1.63) 100.00
NOTE: Weights are from random effects analysis
-18.4
0
18.4

B

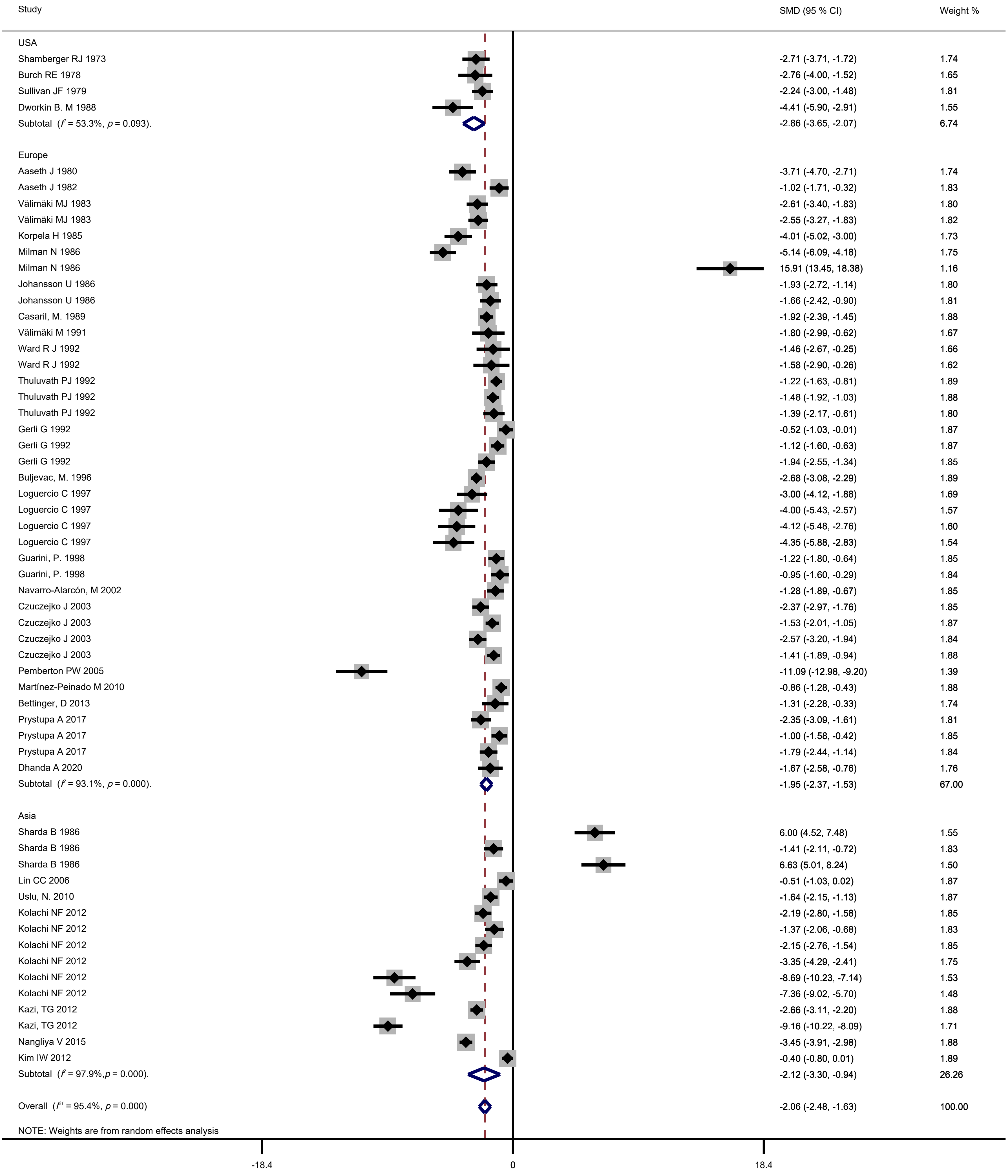
Study
SMD (95 % CI)
Weight %
USA
Shamberger RJ 1973 -2.71 (-3.71, -1.72) 1.74
Burch RE 1978 -2.76 (-4.00, -1.52) 1.65
Sullivan JF 1979 -2.24 (-3.00, -1.48) 1.81
Dworkin B. M 1988 -4.41 (-5.90, -2.91) 1.55
Subtotal (I^2 = 53.3%, p = 0.093). -2.86 (-3.65, -2.07) 6.74
Europe
Aaseth J 1980 -3.71 (-4.70, -2.71) 1.74
Aaseth J 1982 -1.02 (-1.71, -0.32) 1.83
Välimäki MJ 1983 -2.61 (-3.40, -1.83) 1.80
Välimäki MJ 1983 -2.55 (-3.27, -1.83) 1.82
Korpela H 1985 -4.01 (-5.02, -3.00) 1.73
Milman N 1986 -5.14 (-6.09, -4.18) 1.75
Milman N 1986 15.91 (13.45, 18.38) 1.16
Johansson U 1986 -1.93 (-2.72, -1.14) 1.80
Johansson U 1986 -1.66 (-2.42, -0.90) 1.81
Casaril, M. 1989 -1.92 (-2.39, -1.45) 1.88
Välimäki M 1991 -1.80 (-2.99, -0.62) 1.67
Ward R J 1992 -1.46 (-2.67, -0.25) 1.66
Ward R J 1992 -1.58 (-2.90, -0.26) 1.62
Thuluvath PJ 1992 -1.22 (-1.63, -0.81) 1.89
Thuluvath PJ 1992 -1.48 (-1.92, -1.03) 1.88
Thuluvath PJ 1992 -1.39 (-2.17, -0.61) 1.80
Gerli G 1992 -0.52 (-1.03, -0.01) 1.87
Gerli G 1992 -1.12 (-1.60, -0.63) 1.87
Gerli G 1992 -1.94 (-2.55, -1.34) 1.85
Buljevac, M. 1996 -2.68 (-3.08, -2.29) 1.89
Loguercio C 1997 -3.00 (-4.12, -1.88) 1.69
Loguercio C 1997 -4.00 (-5.43, -2.57) 1.57
Loguercio C 1997 -4.12 (-5.48, -2.76) 1.60
Loguercio C 1997 -4.35 (-5.88, -2.83) 1.54
Guarini, P. 1998 -1.22 (-1.80, -0.64) 1.85
Guarini, P. 1998 -0.95 (-1.60, -0.29) 1.84
Navarro-Alarcón, M 2002 -1.28 (-1.89, -0.67) 1.85
Czuczejko J 2003 -2.37 (-2.97, -1.76) 1.85
Czuczejko J 2003 -1.53 (-2.01, -1.05) 1.87
Czuczejko J 2003 -2.57 (-3.20, -1.94) 1.84
Czuczejko J 2003 -1.41 (-1.89, -0.94) 1.88
Pemberton PW 2005 -11.09 (-12.98, -9.20) 1.39
Martínez-Peinado M 2010 -0.86 (-1.28, -0.43) 1.88
Bettinger, D 2013 -1.31 (-2.28, -0.33) 1.74
Prystupa A 2017 -2.35 (-3.09, -1.61) 1.81
Prystupa A 2017 -1.00 (-1.58, -0.42) 1.85
Prystupa A 2017 -1.79 (-2.44, -1.14) 1.84
Dhanda A 2020 -1.67 (-2.58, -0.76) 1.76
Subtotal (I^2 = 93.1%, p = 0.000). -1.95 (-2.37, -1.53) 67.00
Asia
Sharda B 1986 6.00 (4.52, 7.48) 1.55
Sharda B 1986 -1.41 (-2.11, -0.72) 1.83
Sharda B 1986 6.63 (5.01, 8.24) 1.50
Lin CC 2006 -0.51 (-1.03, 0.02) 1.87
Uslu, N. 2010 -1.64 (-2.15, -1.13) 1.87
Kolachi NF 2012 -2.19 (-2.80, -1.58) 1.85
Kolachi NF 2012 -1.37 (-2.06, -0.68) 1.83
Kolachi NF 2012 -2.15 (-2.76, -1.54) 1.85
Kolachi NF 2012 -3.35 (-4.29, -2.41) 1.75
Kolachi NF 2012 -8.69 (-10.23, -7.14) 1.53
Kolachi NF 2012 -7.36 (-9.02, -5.70) 1.48
Kazi, TG 2012 -2.66 (-3.11, -2.20) 1.88
Kazi, TG 2012 -9.16 (-10.22, -8.09) 1.71
Nangliya V 2015 -3.45 (-3.91, -2.98) 1.88
Kim IW 2012 -0.40 (-0.80, 0.01) 1.89
Subtotal (I^2 = 97.9%,p = 0.000). -2.12 (-3.30, -0.94) 26.26
Overall (I^2 = 95.4%, p = 0.000) -2.06 (-2.48, -1.63) 100.00
NOTE: Weights are from random effects analysis
-18.4
0
18.4

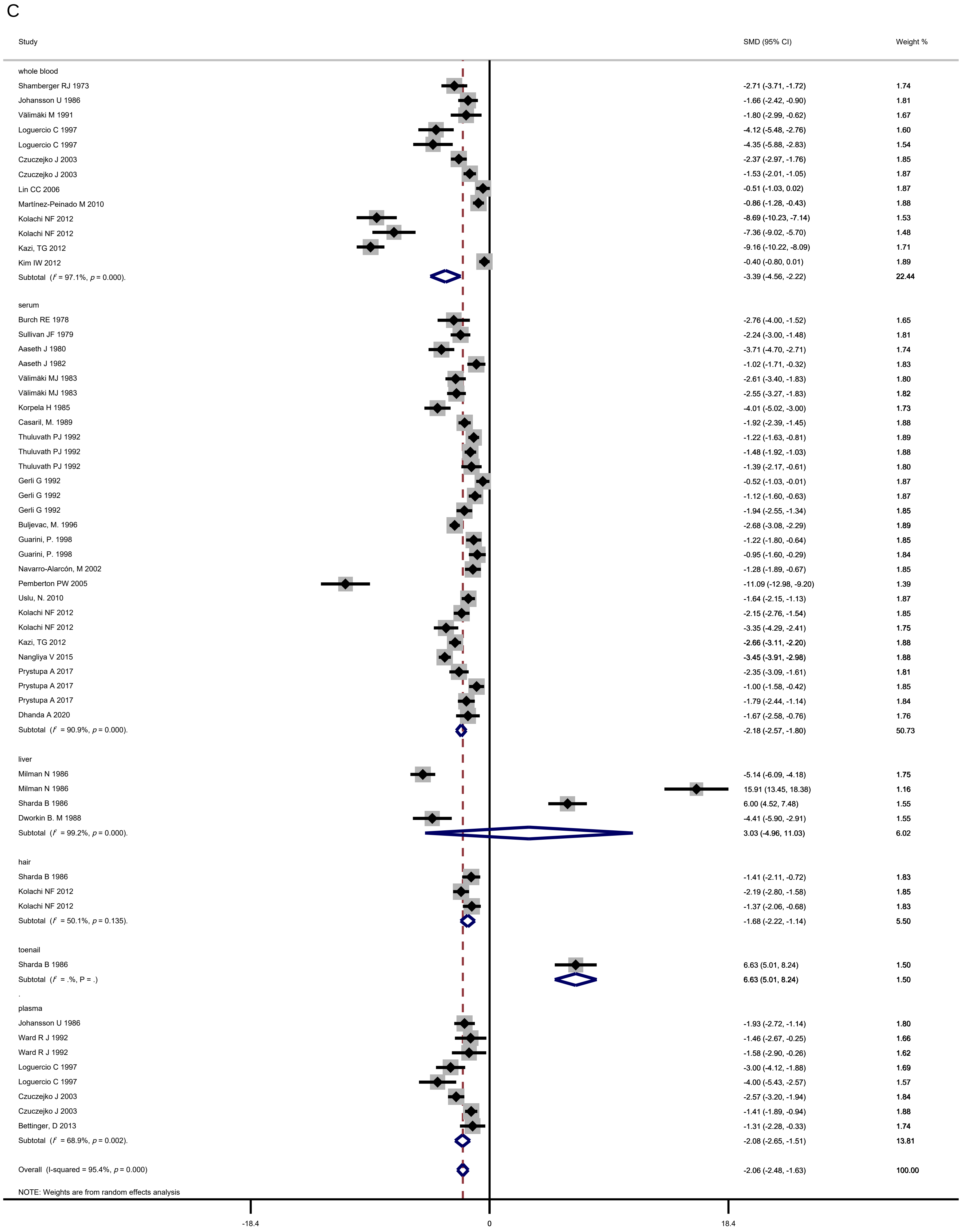
C
Study
SMD (95% CI)
Weight %
whole blood
Shamberger RJ 1973 -2.71 (-3.71, -1.72) 1.74
Johansson U 1986 -1.66 (-2.42, -0.90) 1.81
Välimäki M 1991 -1.80 (-2.99, -0.62) 1.67
Loguercio C 1997 -4.12 (-5.48, -2.76) 1.60
Loguercio C 1997 -4.35 (-5.88, -2.83) 1.54
Czuczejko J 2003 -2.37 (-2.97, -1.76) 1.85
Czuczejko J 2003 -1.53 (-2.01, -1.05) 1.87
Lin CC 2006 -0.51 (-1.03, 0.02) 1.87
Martínez-Peinado M 2010 -0.86 (-1.28, -0.43) 1.88
Kolachi NF 2012 -8.69 (-10.23, -7.14) 1.53
Kolachi NF 2012 -7.36 (-9.02, -5.70) 1.48
Kazi, TG 2012 -9.16 (-10.22, -8.09) 1.71
Kim IW 2012 -0.40 (-0.80, 0.01) 1.89
Subtotal (I^2 = 97.1%, p = 0.000). -3.39 (-4.56, -2.22) 22.44
serum
Burch RE 1978 -2.76 (-4.00, -1.52) 1.65
Sullivan JF 1979 -2.24 (-3.00, -1.48) 1.81
Aaseth J 1980 -3.71 (-4.70, -2.71) 1.74
Aaseth J 1982 -1.02 (-1.71, -0.32) 1.83
Välimäki MJ 1983 -2.61 (-3.40, -1.83) 1.80
Välimäki MJ 1983 -2.55 (-3.27, -1.83) 1.82
Korpela H 1985 -4.01 (-5.02, -3.00) 1.73
Casaril, M. 1989 -1.92 (-2.39, -1.45) 1.88
Thuluvath PJ 1992 -1.22 (-1.63, -0.81) 1.89
Thuluvath PJ 1992 -1.48 (-1.92, -1.03) 1.88
Thuluvath PJ 1992 -1.39 (-2.17, -0.61) 1.80
Gerli G 1992 -0.52 (-1.03, -0.01) 1.87
Gerli G 1992 -1.12 (-1.60, -0.63) 1.87
Gerli G 1992 -1.94 (-2.55, -1.34) 1.85
Buljevac, M. 1996 -2.68 (-3.08, -2.29) 1.89
Guarini, P. 1998 -1.22 (-1.80, -0.64) 1.85
Guarini, P. 1998 -0.95 (-1.60, -0.29) 1.84
Navarro-Alarcón, M 2002 -1.28 (-1.89, -0.67) 1.85
Pemberton PW 2005 -11.09 (-12.98, -9.20) 1.39
Uslu, N. 2010 -1.64 (-2.15, -1.13) 1.87
Kolachi NF 2012 -2.15 (-2.76, -1.54) 1.85
Kolachi NF 2012 -3.35 (-4.29, -2.41) 1.75
Kazi, TG 2012 -2.66 (-3.11, -2.20) 1.88
Nangliya V 2015 -3.45 (-3.91, -2.98) 1.88
Prystupa A 2017 -2.35 (-3.09, -1.61) 1.81
Prystupa A 2017 -1.00 (-1.58, -0.42) 1.85
Prystupa A 2017 -1.79 (-2.44, -1.14) 1.84
Dhanda A 2020 -1.67 (-2.58, -0.76) 1.76
Subtotal (I^2 = 90.9%, p = 0.000). -2.18 (-2.57, -1.80) 50.73
liver
Milman N 1986 -5.14 (-6.09, -4.18) 1.75
Milman N 1986 15.91 (13.45, 18.38) 1.16
Sharda B 1986 6.00 (4.52, 7.48) 1.55
Dworkin B. M 1988 -4.41 (-5.90, -2.91) 1.55
Subtotal (I^2 = 99.2%, p = 0.000). 3.03 (-4.96, 11.03) 6.02
hair
Sharda B 1986 -1.41 (-2.11, -0.72) 1.83
Kolachi NF 2012 -2.19 (-2.80, -1.58) 1.85
Kolachi NF 2012 -1.37 (-2.06, -0.68) 1.83
Subtotal (I^2 = 50.1%, p = 0.135). -1.68 (-2.22, -1.14) 5.50
toenail
Sharda B 1986 6.63 (5.01, 8.24) 1.50
Subtotal (I^2 = .%, P = .) 6.63 (5.01, 8.24) 1.50
.
plasma
Johansson U 1986 -1.93 (-2.72, -1.14) 1.80
Ward R J 1992 -1.46 (-2.67, -0.25) 1.66
Ward R J 1992 -1.58 (-2.90, -0.26) 1.62
Loguercio C 1997 -3.00 (-4.12, -1.88) 1.69
Loguercio C 1997 -4.00 (-5.43, -2.57) 1.57
Czuczejko J 2003 -2.57 (-3.20, -1.94) 1.84
Czuczejko J 2003 -1.41 (-1.89, -0.94) 1.88
Bettinger, D 2013 -1.31 (-2.28, -0.33) 1.74
Subtotal (I^2 = 68.9%, p = 0.002). -2.08 (-2.65, -1.51) 13.81
Overall (I-squared = 95.4%, p = 0.000) -2.06 (-2.48, -1.63) 100.00
NOTE: Weights are from random effects analysis
-18.4
0
18.4

D

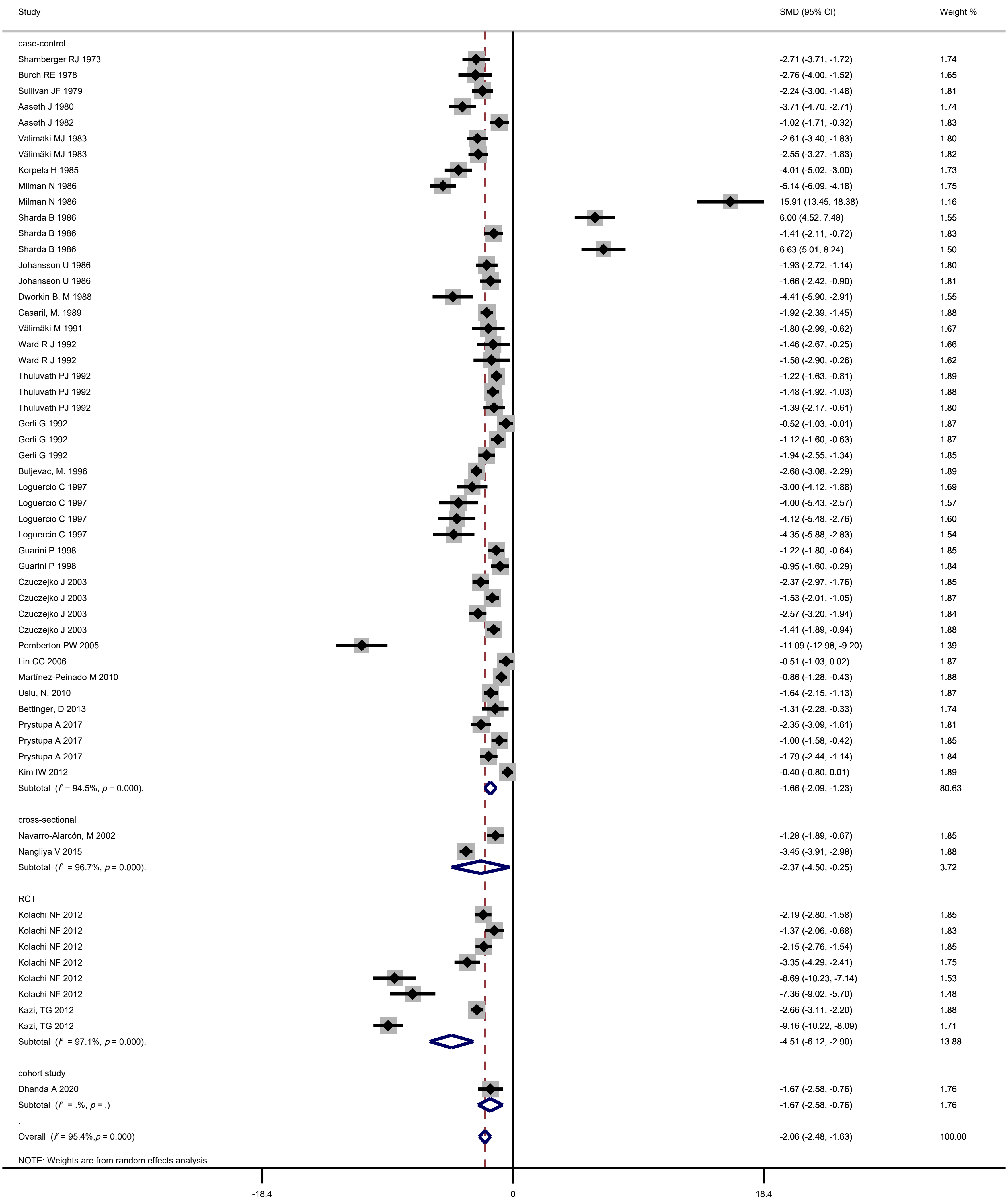

Study
SMD (95% CI)
Weight %
case-control
Shamberger RJ 1973 -2.71 (-3.71, -1.72) 1.74
Burch RE 1978 -2.76 (-4.00, -1.52) 1.65
Sullivan JF 1979 -2.24 (-3.00, -1.48) 1.81
Aaseth J 1980 -3.71 (-4.70, -2.71) 1.74
Aaseth J 1982 -1.02 (-1.71, -0.32) 1.83
Välimäki MJ 1983 -2.61 (-3.40, -1.83) 1.80
Välimäki MJ 1983 -2.55 (-3.27, -1.83) 1.82
Korpela H 1985 -4.01 (-5.02, -3.00) 1.73
Milman N 1986 -5.14 (-6.09, -4.18) 1.75
Milman N 1986 15.91 (13.45, 18.38) 1.16
Sharda B 1986 6.00 (4.52, 7.48) 1.55
Sharda B 1986 -1.41 (-2.11, -0.72) 1.83
Sharda B 1986 6.63 (5.01, 8.24) 1.50
Johansson U 1986 -1.93 (-2.72, -1.14) 1.80
Johansson U 1986 -1.66 (-2.42, -0.90) 1.81
Dworkin B. M 1988 -4.41 (-5.90, -2.91) 1.55
Casaril, M. 1989 -1.92 (-2.39, -1.45) 1.88
Välimäki M 1991 -1.80 (-2.99, -0.62) 1.67
Ward R J 1992 -1.46 (-2.67, -0.25) 1.66
Ward R J 1992 -1.58 (-2.90, -0.26) 1.62
Thuluvath PJ 1992 -1.22 (-1.63, -0.81) 1.89
Thuluvath PJ 1992 -1.48 (-1.92, -1.03) 1.88
Thuluvath PJ 1992 -1.39 (-2.17, -0.61) 1.80
Gerli G 1992 -0.52 (-1.03, -0.01) 1.87
Gerli G 1992 -1.12 (-1.60, -0.63) 1.87
Gerli G 1992 -1.94 (-2.55, -1.34) 1.85
Buljevac, M. 1996 -2.68 (-3.08, -2.29) 1.89
Loguercio C 1997 -3.00 (-4.12, -1.88) 1.69
Loguercio C 1997 -4.00 (-5.43, -2.57) 1.57
Loguercio C 1997 -4.12 (-5.48, -2.76) 1.60
Loguercio C 1997 -4.35 (-5.88, -2.83) 1.54
Guarini P 1998 -1.22 (-1.80, -0.64) 1.85
Guarini P 1998 -0.95 (-1.60, -0.29) 1.84
Czuczejko J 2003 -2.37 (-2.97, -1.76) 1.85
Czuczejko J 2003 -1.53 (-2.01, -1.05) 1.87
Czuczejko J 2003 -2.57 (-3.20, -1.94) 1.84
Czuczejko J 2003 -1.41 (-1.89, -0.94) 1.88
Pemberton PW 2005 -11.09 (-12.98, -9.20) 1.39
Lin CC 2006 -0.51 (-1.03, 0.02) 1.87
Martínez-Peinado M 2010 -0.86 (-1.28, -0.43) 1.88
Uslu, N. 2010 -1.64 (-2.15, -1.13) 1.87
Bettinger, D 2013 -1.31 (-2.28, -0.33) 1.74
Prystupa A 2017 -2.35 (-3.09, -1.61) 1.81
Prystupa A 2017 -1.00 (-1.58, -0.42) 1.85
Prystupa A 2017 -1.79 (-2.44, -1.14) 1.84
Kim IW 2012 -0.40 (-0.80, 0.01) 1.89
Subtotal (I^2 = 94.5%, p = 0.000). -1.66 (-2.09, -1.23) 80.63
cross-sectional
Navarro-Alarcón, M 2002 -1.28 (-1.89, -0.67) 1.85
Nangliya V 2015 -3.45 (-3.91, -2.98) 1.88
Subtotal (I^2 = 96.7%, p = 0.000). -2.37 (-4.50, -0.25) 3.72
RCT
Kolachi NF 2012 -2.19 (-2.80, -1.58) 1.85
Kolachi NF 2012 -1.37 (-2.06, -0.68) 1.83
Kolachi NF 2012 -2.15 (-2.76, -1.54) 1.85
Kolachi NF 2012 -3.35 (-4.29, -2.41) 1.75
Kolachi NF 2012 -8.69 (-10.23, -7.14) 1.53
Kolachi NF 2012 -7.36 (-9.02, -5.70) 1.48
Kazi, TG 2012 -2.66 (-3.11, -2.20) 1.88
Kazi, TG 2012 -9.16 (-10.22, -8.09) 1.71
Subtotal (I^2 = 97.1%, p = 0.000). -4.51 (-6.12, -2.90) 13.88
cohort study
Dhanda A 2020 -1.67 (-2.58, -0.76) 1.76
Subtotal (I^2 = .%, p = .) -1.67 (-2.58, -0.76) 1.76
.
Overall (I^2 = 95.4%, p = 0.000) -2.06 (-2.48, -1.63) 100.00
NOTE: Weights are from random effects analysis
-18.4
0
18.4

E

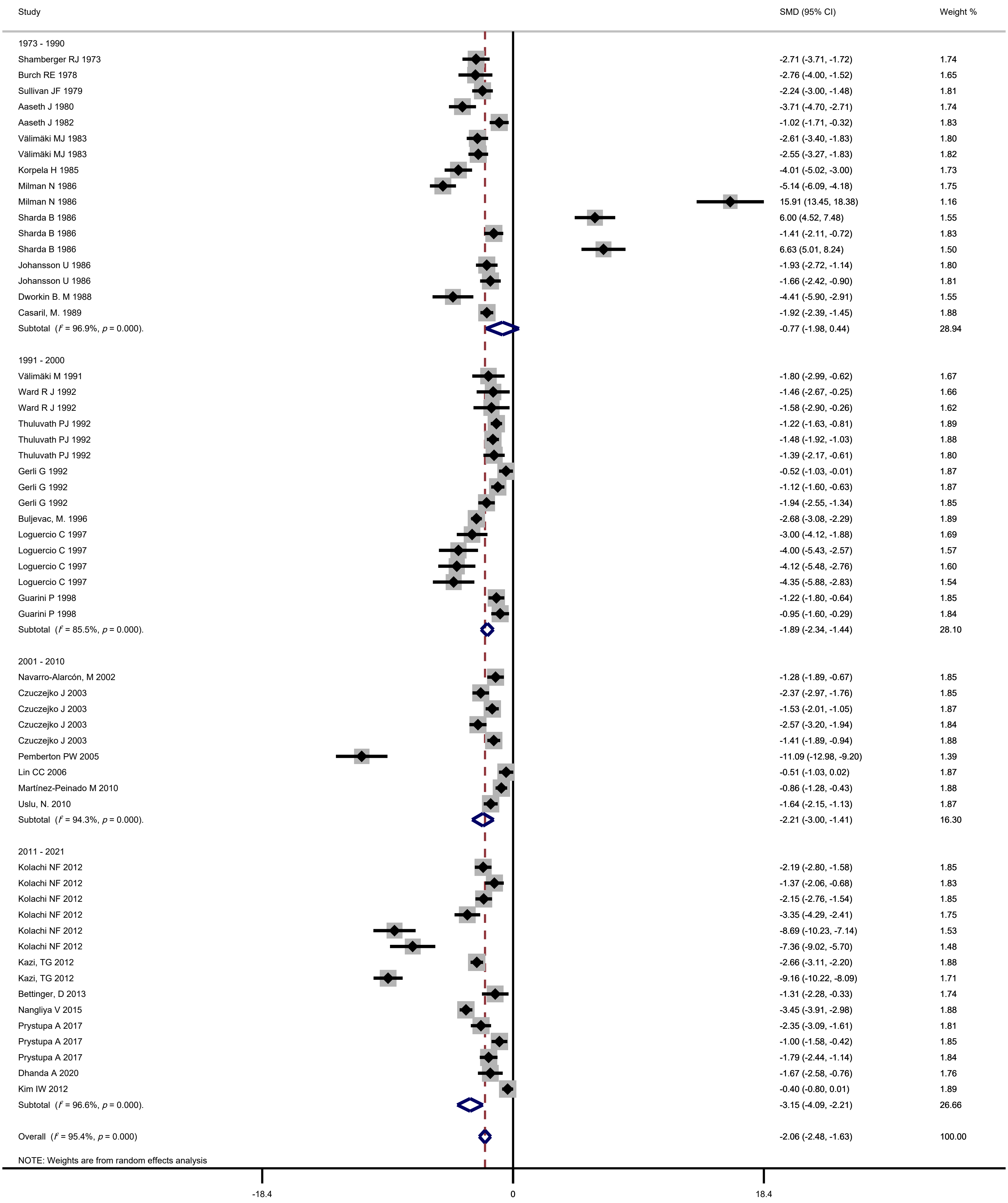

Study
SMD (95% CI)
Weight %
1973 - 1990
Shamberger RJ 1973 -2.71 (-3.71, -1.72) 1.74
Burch RE 1978 -2.76 (-4.00, -1.52) 1.65
Sullivan JF 1979 -2.24 (-3.00, -1.48) 1.81
Aaseth J 1980 -3.71 (-4.70, -2.71) 1.74
Aaseth J 1982 -1.02 (-1.71, -0.32) 1.83
Välimäki MJ 1983 -2.61 (-3.40, -1.83) 1.80
Välimäki MJ 1983 -2.55 (-3.27, -1.83) 1.82
Korpela H 1985 -4.01 (-5.02, -3.00) 1.73
Milman N 1986 -5.14 (-6.09, -4.18) 1.75
Milman N 1986 15.91 (13.45, 18.38) 1.16
Sharda B 1986 6.00 (4.52, 7.48) 1.55
Sharda B 1986 -1.41 (-2.11, -0.72) 1.83
Sharda B 1986 6.63 (5.01, 8.24) 1.50
Johansson U 1986 -1.93 (-2.72, -1.14) 1.80
Johansson U 1986 -1.66 (-2.42, -0.90) 1.81
Dworkin B. M 1988 -4.41 (-5.90, -2.91) 1.55
Casaril, M. 1989 -1.92 (-2.39, -1.45) 1.88
Subtotal (I^2 = 96.9%, p = 0.000). -0.77 (-1.98, 0.44) 28.94
1991 - 2000
Välimäki M 1991 -1.80 (-2.99, -0.62) 1.67
Ward R J 1992 -1.46 (-2.67, -0.25) 1.66
Ward R J 1992 -1.58 (-2.90, -0.26) 1.62
Thuluvath PJ 1992 -1.22 (-1.63, -0.81) 1.89
Thuluvath PJ 1992 -1.48 (-1.92, -1.03) 1.88
Thuluvath PJ 1992 -1.39 (-2.17, -0.61) 1.80
Gerli G 1992 -0.52 (-1.03, -0.01) 1.87
Gerli G 1992 -1.12 (-1.60, -0.63) 1.87
Gerli G 1992 -1.94 (-2.55, -1.34) 1.85
Buljevac, M. 1996 -2.68 (-3.08, -2.29) 1.89
Loguercio C 1997 -3.00 (-4.12, -1.88) 1.69
Loguercio C 1997 -4.00 (-5.43, -2.57) 1.57
Loguercio C 1997 -4.12 (-5.48, -2.76) 1.60
Loguercio C 1997 -4.35 (-5.88, -2.83) 1.54
Guarini P 1998 -1.22 (-1.80, -0.64) 1.85
Guarini P 1998 -0.95 (-1.60, -0.29) 1.84
Subtotal (I^2 = 85.5%, p = 0.000). -1.89 (-2.34, -1.44) 28.10
2001 - 2010
Navarro-Alarcón, M 2002 -1.28 (-1.89, -0.67) 1.85
Czuczejko J 2003 -2.37 (-2.97, -1.76) 1.85
Czuczejko J 2003 -1.53 (-2.01, -1.05) 1.87
Czuczejko J 2003 -2.57 (-3.20, -1.94) 1.84
Czuczejko J 2003 -1.41 (-1.89, -0.94) 1.88
Pemberton PW 2005 -11.09 (-12.98, -9.20) 1.39
Lin CC 2006 -0.51 (-1.03, 0.02) 1.87
Martínez-Peinado M 2010 -0.86 (-1.28, -0.43) 1.88
Uslu, N. 2010 -1.64 (-2.15, -1.13) 1.87
Subtotal (I^2 = 94.3%, p = 0.000). -2.21 (-3.00, -1.41) 16.30
2011 - 2021
Kolachi NF 2012 -2.19 (-2.80, -1.58) 1.85
Kolachi NF 2012 -1.37 (-2.06, -0.68) 1.83
Kolachi NF 2012 -2.15 (-2.76, -1.54) 1.85
Kolachi NF 2012 -3.35 (-4.29, -2.41) 1.75
Kolachi NF 2012 -8.69 (-10.23, -7.14) 1.53
Kolachi NF 2012 -7.36 (-9.02, -5.70) 1.48
Kazi, TG 2012 -2.66 (-3.11, -2.20) 1.88
Kazi, TG 2012 -9.16 (-10.22, -8.09) 1.71
Bettinger, D 2013 -1.31 (-2.28, -0.33) 1.74
Nangliya V 2015 -3.45 (-3.91, -2.98) 1.88
Prystupa A 2017 -2.35 (-3.09, -1.61) 1.81
Prystupa A 2017 -1.00 (-1.58, -0.42) 1.85
Prystupa A 2017 -1.79 (-2.44, -1.14) 1.84
Dhanda A 2020 -1.67 (-2.58, -0.76) 1.76
Kim IW 2012 -0.40 (-0.80, 0.01) 1.89
Subtotal (I^2 = 96.6%, p = 0.000). -3.15 (-4.09, -2.21) 26.66
Overall (I^2 = 95.4%, p = 0.000) -2.06 (-2.48, -1.63) 100.00
NOTE: Weights are from random effects analysis
-18.4
0
18.4

F

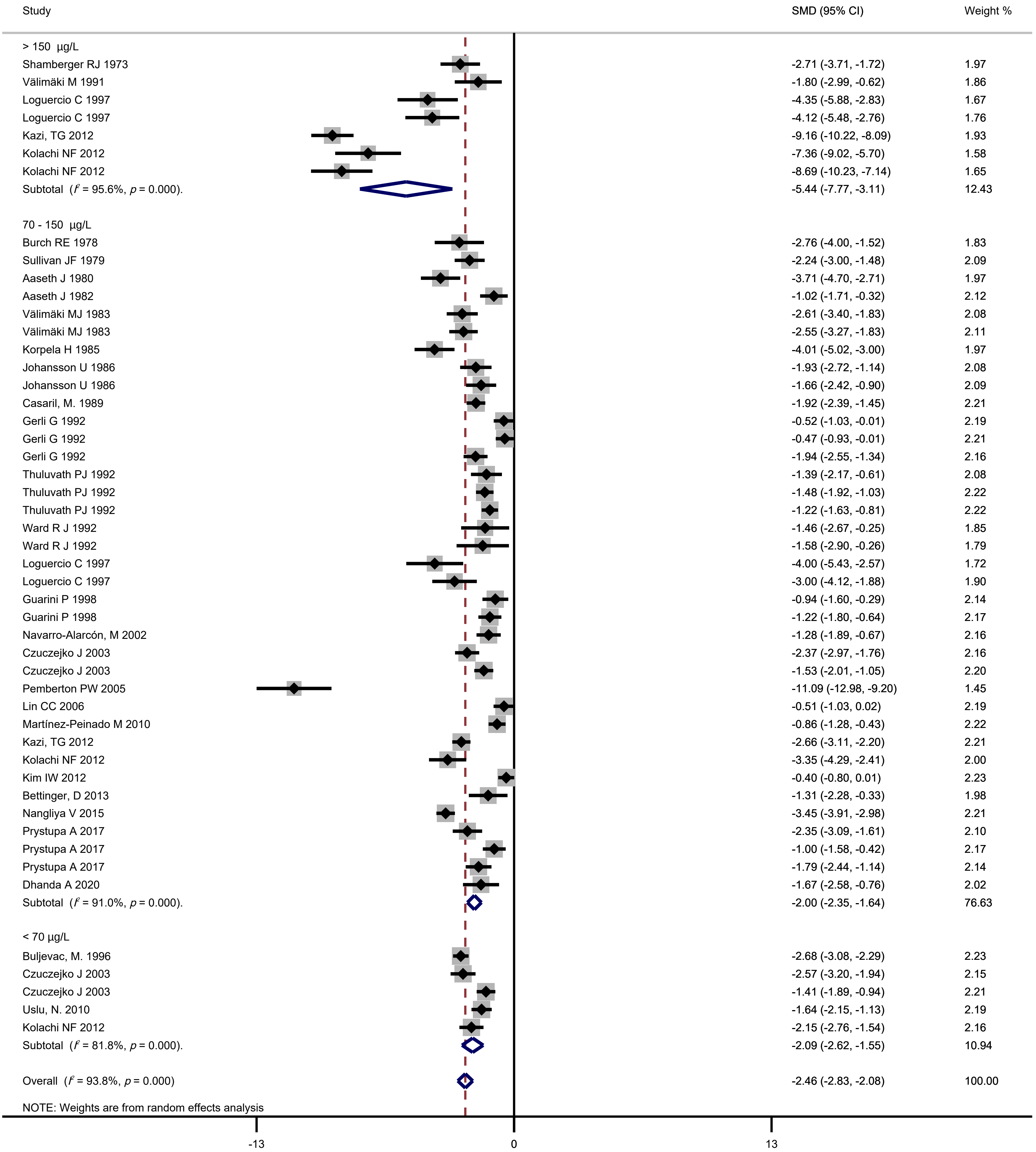

Study
SMD (95% CI)
Weight %
> 150 µg/L
Shamberger RJ 1973 -2.71 (-3.71, -1.72) 1.97
Välimäki M 1991 -1.80 (-2.99, -0.62) 1.86
Loguercio C 1997 -4.35 (-5.88, -2.83) 1.67
Loguercio C 1997 -4.12 (-5.48, -2.76) 1.76
Kazi, TG 2012 -9.16 (-10.22, -8.09) 1.93
Kolachi NF 2012 -7.36 (-9.02, -5.70) 1.58
Kolachi NF 2012 -8.69 (-10.23, -7.14) 1.65
Subtotal (I^2 = 95.6%, p = 0.000). -5.44 (-7.77, -3.11) 12.43
70 - 150 µg/L
Burch RE 1978 -2.76 (-4.00, -1.52) 1.83
Sullivan JF 1979 -2.24 (-3.00, -1.48) 2.09
Aaseth J 1980 -3.71 (-4.70, -2.71) 1.97
Aaseth J 1982 -1.02 (-1.71, -0.32) 2.12
Välimäki MJ 1983 -2.61 (-3.40, -1.83) 2.08
Välimäki MJ 1983 -2.55 (-3.27, -1.83) 2.11
Korpela H 1985 -4.01 (-5.02, -3.00) 1.97
Johansson U 1986 -1.93 (-2.72, -1.14) 2.08
Johansson U 1986 -1.66 (-2.42, -0.90) 2.09
Casaril, M. 1989 -1.92 (-2.39, -1.45) 2.21
Gerli G 1992 -0.52 (-1.03, -0.01) 2.19
Gerli G 1992 -0.47 (-0.93, -0.01) 2.21
Gerli G 1992 -1.94 (-2.55, -1.34) 2.16
Thuluvath PJ 1992 -1.39 (-2.17, -0.61) 2.08
Thuluvath PJ 1992 -1.48 (-1.92, -1.03) 2.22
Thuluvath PJ 1992 -1.22 (-1.63, -0.81) 2.22
Ward R J 1992 -1.46 (-2.67, -0.25) 1.85
Ward R J 1992 -1.58 (-2.90, -0.26) 1.79
Loguercio C 1997 -4.00 (-5.43, -2.57) 1.72
Loguercio C 1997 -3.00 (-4.12, -1.88) 1.90
Guarini P 1998 -0.94 (-1.60, -0.29) 2.14
Guarini P 1998 -1.22 (-1.80, -0.64) 2.17
Navarro-Alarcón, M 2002 -1.28 (-1.89, -0.67) 2.16
Czuczejko J 2003 -2.37 (-2.97, -1.76) 2.16
Czuczejko J 2003 -1.53 (-2.01, -1.05) 2.20
Pemberton PW 2005 -11.09 (-12.98, -9.20) 1.45
Lin CC 2006 -0.51 (-1.03, 0.02) 2.19
Martínez-Peinado M 2010 -0.86 (-1.28, -0.43) 2.22
Kazi, TG 2012 -2.66 (-3.11, -2.20) 2.21
Kolachi NF 2012 -3.35 (-4.29, -2.41) 2.00
Kim IW 2012 -0.40 (-0.80, 0.01) 2.23
Bettinger, D 2013 -1.31 (-2.28, -0.33) 1.98
Nangliya V 2015 -3.45 (-3.91, -2.98) 2.21
Prystupa A 2017 -2.35 (-3.09, -1.61) 2.10
Prystupa A 2017 -1.00 (-1.58, -0.42) 2.17
Prystupa A 2017 -1.79 (-2.44, -1.14) 2.14
Dhanda A 2020 -1.67 (-2.58, -0.76) 2.02
Subtotal (I^2 = 91.0%, p = 0.000). -2.00 (-2.35, -1.64) 76.63
< 70 µg/L
Buljevac, M. 1996 -2.68 (-3.08, -2.29) 2.23
Czuczejko J 2003 -2.57 (-3.20, -1.94) 2.15
Czuczejko J 2003 -1.41 (-1.89, -0.94) 2.21
Uslu, N. 2010 -1.64 (-2.15, -1.13) 2.19
Kolachi NF 2012 -2.15 (-2.76, -1.54) 2.16
Subtotal (I^2 = 81.8%, p = 0.000). -2.09 (-2.62, -1.55) 10.94
Overall (I^2 = 93.8%, p = 0.000) -2.46 (-2.83, -2.08) 100.00
NOTE: Weights are from random effects analysis
-13
0
13

Figure S4. Forest plot in the meta-analysis of selenium levels and cirrhosis. The dot represents the effect size of each study. The line through the dot represents 95% CI of the effect size[13,30,32,34 − 39,41,42,44,48,50,52,54 − 70]. The size of the square represents the weight, Wi. The broken line represents the effect size and the horizontal length of the diamond the 95% CI of the effect size for all studies combined. (A) stratified by disease types;(B) stratified by study regions; (C) stratified by sample sources; (D) stratified by study design; (E) stratified by year of publication; (F) stratified by the reference blood (include whole blood, serum, plasma) selenium levels (70-150 μg/L).

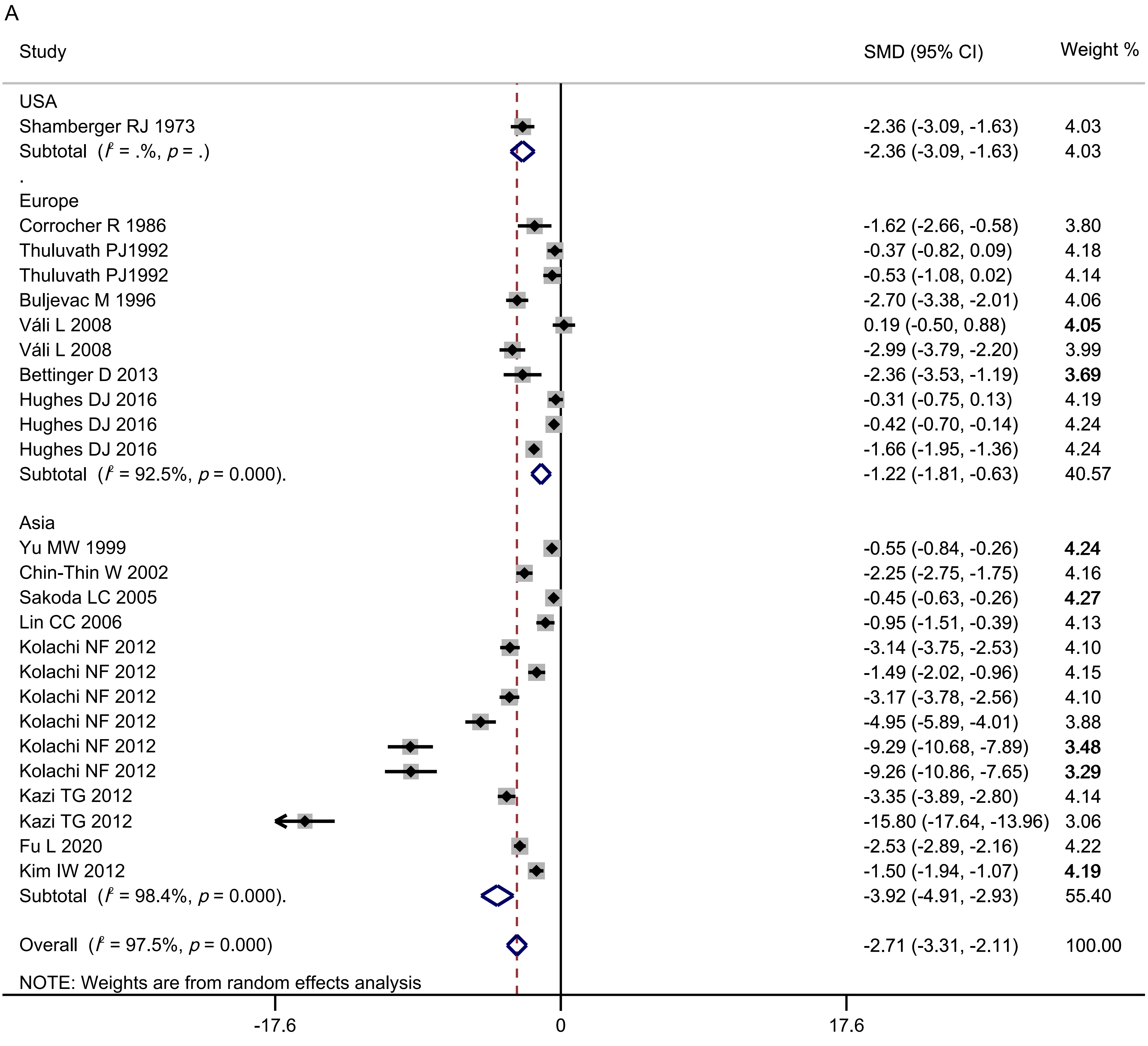
A
Study
SMD (95% CI)
Weight %
USA
Shamberger RJ 1973 -2.36 (-3.09, -1.63) 4.03
Subtotal (I^2 = .%, p = .) -2.36 (-3.09, -1.63) 4.03
.
Europe
Corrocher R 1986 -1.62 (-2.66, -0.58) 3.80
Thuluvath PJ1992 -0.37 (-0.82, 0.09) 4.18
Thuluvath PJ1992 -0.53 (-1.08, 0.02) 4.14
Buljevac M 1996 -2.70 (-3.38, -2.01) 4.06
Váli L 2008 0.19 (-0.50, 0.88) **4.05**
Váli L 2008 -2.99 (-3.79, -2.20) 3.99
Bettinger D 2013 -2.36 (-3.53, -1.19) **3.69**
Hughes DJ 2016 -0.31 (-0.75, 0.13) 4.19
Hughes DJ 2016 -0.42 (-0.70, -0.14) 4.24
Hughes DJ 2016 -1.66 (-1.95, -1.36) 4.24
Subtotal (I^2 = 92.5%, p = 0.000). -1.22 (-1.81, -0.63) 40.57
Asia
Yu MW 1999 -0.55 (-0.84, -0.26) **4.24**
Chin-Thin W 2002 -2.25 (-2.75, -1.75) 4.16
Sakoda LC 2005 -0.45 (-0.63, -0.26) **4.27**
Lin CC 2006 -0.95 (-1.51, -0.39) 4.13
Kolachi NF 2012 -3.14 (-3.75, -2.53) 4.10
Kolachi NF 2012 -1.49 (-2.02, -0.96) 4.15
Kolachi NF 2012 -3.17 (-3.78, -2.56) 4.10
Kolachi NF 2012 -4.95 (-5.89, -4.01) 3.88
Kolachi NF 2012 -9.29 (-10.68, -7.89) **3.48**
Kolachi NF 2012 -9.26 (-10.86, -7.65) **3.29**
Kazi TG 2012 -3.35 (-3.89, -2.80) 4.14
Kazi TG 2012 -15.80 (-17.64, -13.96) 3.06
Fu L 2020 -2.53 (-2.89, -2.16) 4.22
Kim IW 2012 -1.50 (-1.94, -1.07) **4.19**
Subtotal (I^2 = 98.4%, p = 0.000). -3.92 (-4.91, -2.93) 55.40
Overall (I^2 = 97.5%, p = 0.000) -2.71 (-3.31, -2.11) 100.00
NOTE: Weights are from random effects analysis
-17.6
0
17.6

B

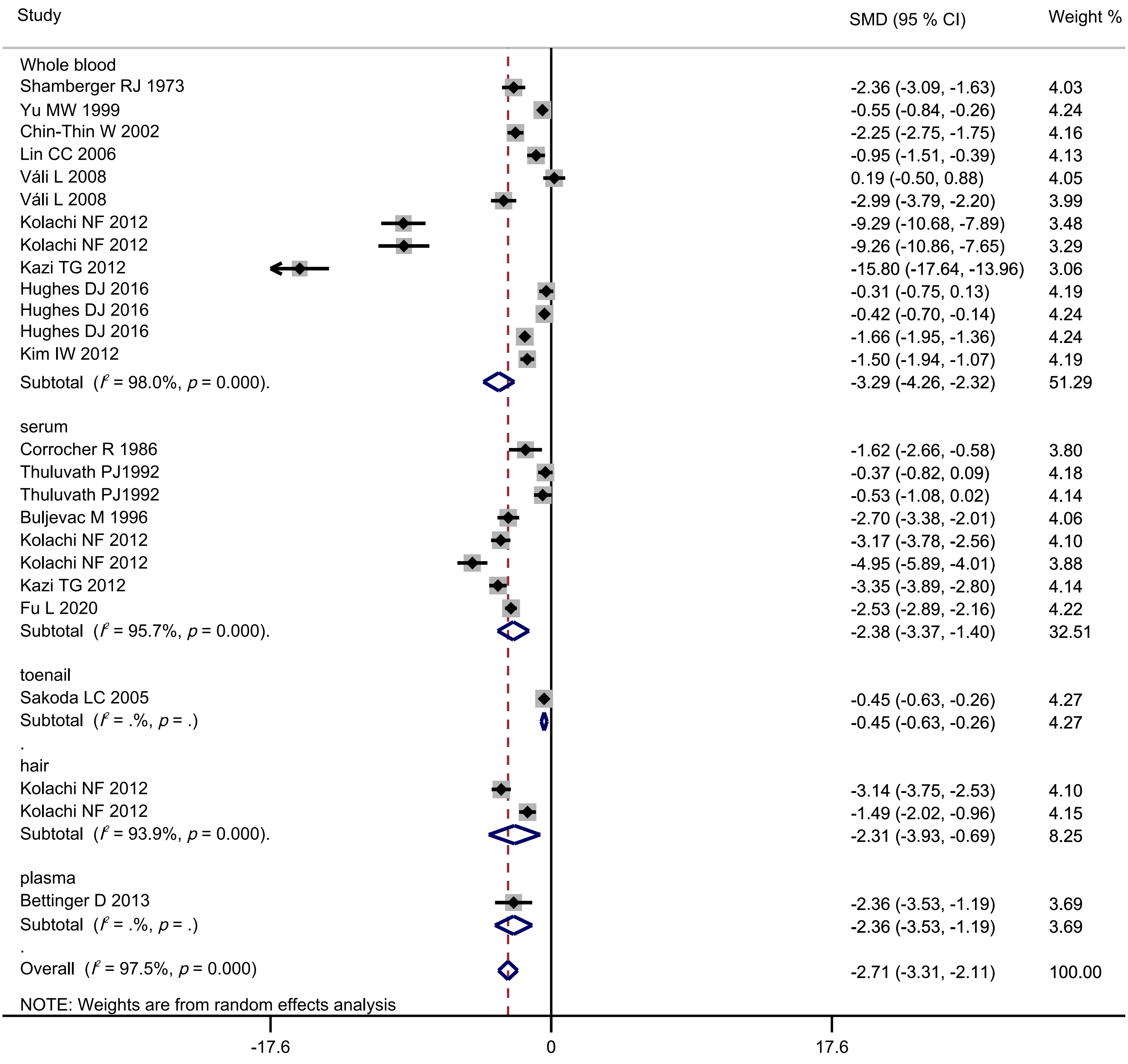
Study
SMD (95 % CI)
Weight %
Whole blood
Shamberger RJ 1973 -2.36 (-3.09, -1.63) 4.03
Yu MW 1999 -0.55 (-0.84, -0.26) 4.24
Chin-Thin W 2002 -2.25 (-2.75, -1.75) 4.16
Lin CC 2006 -0.95 (-1.51, -0.39) 4.13
Váli L 2008 0.19 (-0.50, 0.88) 4.05
Váli L 2008 -2.99 (-3.79, -2.20) 3.99
Kolachi NF 2012 -9.29 (-10.68, -7.89) 3.48
Kolachi NF 2012 -9.26 (-10.86, -7.65) 3.29
Kazi TG 2012 -15.80 (-17.64, -13.96) 3.06
Hughes DJ 2016 -0.31 (-0.75, 0.13) 4.19
Hughes DJ 2016 -0.42 (-0.70, -0.14) 4.24
Hughes DJ 2016 -1.66 (-1.95, -1.36) 4.24
Kim IW 2012 -1.50 (-1.94, -1.07) 4.19
Subtotal (I^2 = 98.0%, p = 0.000). -3.29 (-4.26, -2.32) 51.29
serum
Corrocher R 1986 -1.62 (-2.66, -0.58) 3.80
Thuluvath PJ1992 -0.37 (-0.82, 0.09) 4.18
Thuluvath PJ1992 -0.53 (-1.08, 0.02) 4.14
Buljevac M 1996 -2.70 (-3.38, -2.01) 4.06
Kolachi NF 2012 -3.17 (-3.78, -2.56) 4.10
Kolachi NF 2012 -4.95 (-5.89, -4.01) 3.88
Kazi TG 2012 -3.35 (-3.89, -2.80) 4.14
Fu L 2020 -2.53 (-2.89, -2.16) 4.22
Subtotal (I^2 = 95.7%, p = 0.000). -2.38 (-3.37, -1.40) 32.51
toenail
Sakoda LC 2005 -0.45 (-0.63, -0.26) 4.27
Subtotal (I^2 = .%, p = .) -0.45 (-0.63, -0.26) 4.27
.
hair
Kolachi NF 2012 -3.14 (-3.75, -2.53) 4.10
Kolachi NF 2012 -1.49 (-2.02, -0.96) 4.15
Subtotal (I^2 = 93.9%, p = 0.000). -2.31 (-3.93, -0.69) 8.25
plasma
Bettinger D 2013 -2.36 (-3.53, -1.19) 3.69
Subtotal (I^2 = .%, p = .) -2.36 (-3.53, -1.19) 3.69
.
Overall (I^2 = 97.5%, p = 0.000) -2.71 (-3.31, -2.11) 100.00
NOTE: Weights are from random effects analysis
-17.6
0
17.6

C

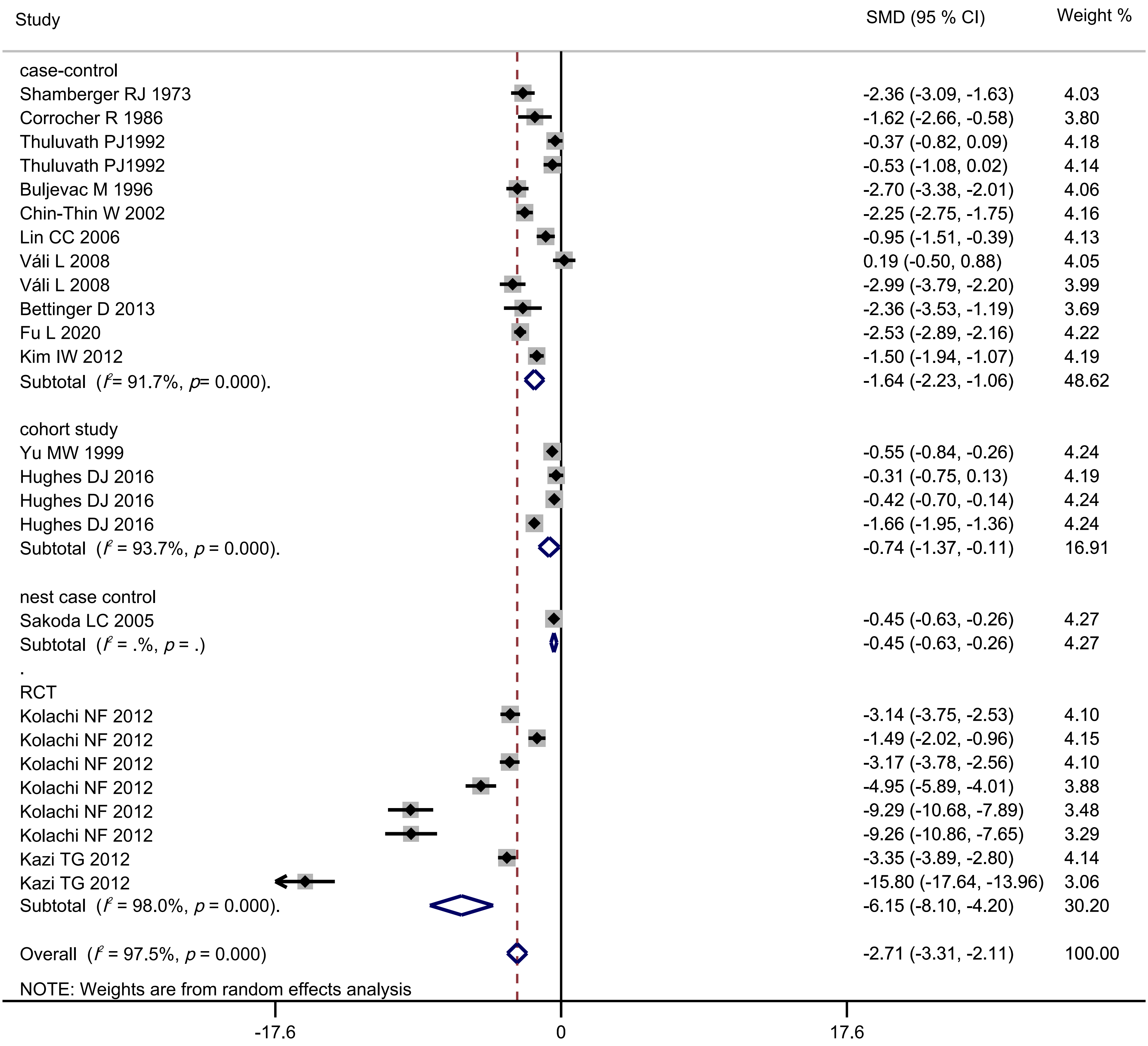
Study
SMD (95 % CI)
Weight %
case-control
Shamberger RJ 1973 -2.36 (-3.09, -1.63) 4.03
Corrocher R 1986 -1.62 (-2.66, -0.58) 3.80
Thuluvath PJ1992 -0.37 (-0.82, 0.09) 4.18
Thuluvath PJ1992 -0.53 (-1.08, 0.02) 4.14
Buljevac M 1996 -2.70 (-3.38, -2.01) 4.06
Chin-Thin W 2002 -2.25 (-2.75, -1.75) 4.16
Lin CC 2006 -0.95 (-1.51, -0.39) 4.13
Váli L 2008 0.19 (-0.50, 0.88) 4.05
Váli L 2008 -2.99 (-3.79, -2.20) 3.99
Bettinger D 2013 -2.36 (-3.53, -1.19) 3.69
Fu L 2020 -2.53 (-2.89, -2.16) 4.22
Kim IW 2012 -1.50 (-1.94, -1.07) 4.19
Subtotal (I^2= 91.7%, p= 0.000). -1.64 (-2.23, -1.06) 48.62
cohort study
Yu MW 1999 -0.55 (-0.84, -0.26) 4.24
Hughes DJ 2016 -0.31 (-0.75, 0.13) 4.19
Hughes DJ 2016 -0.42 (-0.70, -0.14) 4.24
Hughes DJ 2016 -1.66 (-1.95, -1.36) 4.24
Subtotal (I^2 = 93.7%, p = 0.000). -0.74 (-1.37, -0.11) 16.91
nest case control
Sakoda LC 2005 -0.45 (-0.63, -0.26) 4.27
Subtotal (I^2 = .%, p = .) -0.45 (-0.63, -0.26) 4.27
.
RCT
Kolachi NF 2012 -3.14 (-3.75, -2.53) 4.10
Kolachi NF 2012 -1.49 (-2.02, -0.96) 4.15
Kolachi NF 2012 -3.17 (-3.78, -2.56) 4.10
Kolachi NF 2012 -4.95 (-5.89, -4.01) 3.88
Kolachi NF 2012 -9.29 (-10.68, -7.89) 3.48
Kolachi NF 2012 -9.26 (-10.86, -7.65) 3.29
Kazi TG 2012 -3.35 (-3.89, -2.80) 4.14
Kazi TG 2012 -15.80 (-17.64, -13.96) 3.06
Subtotal (I^2 = 98.0%, p = 0.000). -6.15 (-8.10, -4.20) 30.20
Overall (I^2 = 97.5%, p = 0.000) -2.71 (-3.31, -2.11) 100.00
NOTE: Weights are from random effects analysis
-17.6
0
17.6

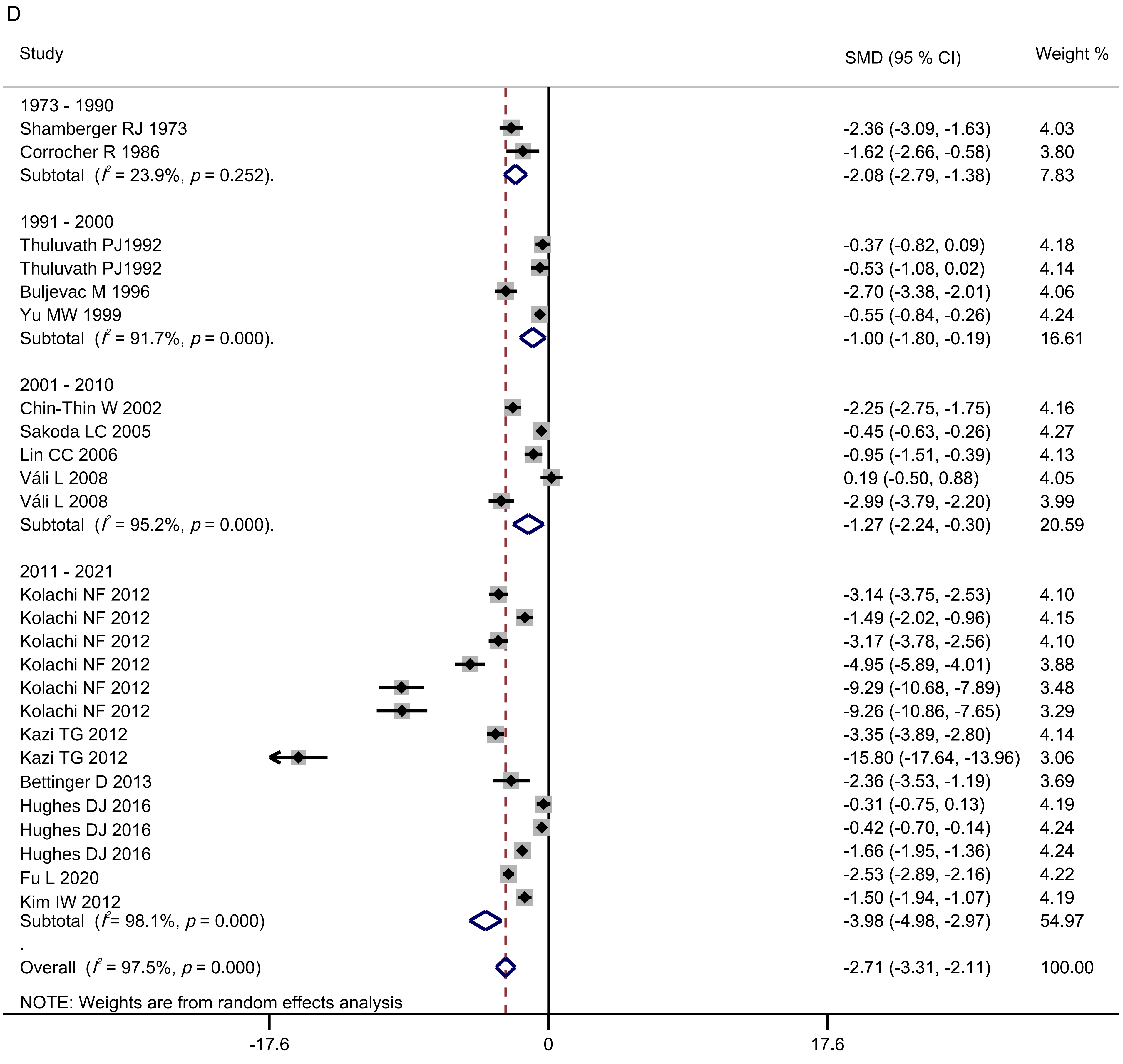
D
Study
SMD (95 % CI)
Weight %
1973 - 1990
Shamberger RJ 1973 -2.36 (-3.09, -1.63) 4.03
Corrocher R 1986 -1.62 (-2.66, -0.58) 3.80
Subtotal (I^2 = 23.9%, p = 0.252). -2.08 (-2.79, -1.38) 7.83
1991 - 2000
Thuluvath PJ1992 -0.37 (-0.82, 0.09) 4.18
Thuluvath PJ1992 -0.53 (-1.08, 0.02) 4.14
Buljevac M 1996 -2.70 (-3.38, -2.01) 4.06
Yu MW 1999 -0.55 (-0.84, -0.26) 4.24
Subtotal (I^2 = 91.7%, p = 0.000). -1.00 (-1.80, -0.19) 16.61
2001 - 2010
Chin-Thin W 2002 -2.25 (-2.75, -1.75) 4.16
Sakoda LC 2005 -0.45 (-0.63, -0.26) 4.27
Lin CC 2006 -0.95 (-1.51, -0.39) 4.13
Váli L 2008 0.19 (-0.50, 0.88) 4.05
Váli L 2008 -2.99 (-3.79, -2.20) 3.99
Subtotal (I^2 = 95.2%, p = 0.000). -1.27 (-2.24, -0.30) 20.59
2011 - 2021
Kolachi NF 2012 -3.14 (-3.75, -2.53) 4.10
Kolachi NF 2012 -1.49 (-2.02, -0.96) 4.15
Kolachi NF 2012 -3.17 (-3.78, -2.56) 4.10
Kolachi NF 2012 -4.95 (-5.89, -4.01) 3.88
Kolachi NF 2012 -9.29 (-10.68, -7.89) 3.48
Kolachi NF 2012 -9.26 (-10.86, -7.65) 3.29
Kazi TG 2012 -3.35 (-3.89, -2.80) 4.14
Kazi TG 2012 -15.80 (-17.64, -13.96) 3.06
Bettinger D 2013 -2.36 (-3.53, -1.19) 3.69
Hughes DJ 2016 -0.31 (-0.75, 0.13) 4.19
Hughes DJ 2016 -0.42 (-0.70, -0.14) 4.24
Hughes DJ 2016 -1.66 (-1.95, -1.36) 4.24
Fu L 2020 -2.53 (-2.89, -2.16) 4.22
Kim IW 2012 -1.50 (-1.94, -1.07) 4.19
Subtotal (I^2= 98.1%, p = 0.000) -3.98 (-4.98, -2.97) 54.97
.
Overall (I^2 = 97.5%, p = 0.000) -2.71 (-3.31, -2.11) 100.00
NOTE: Weights are from random effects analysis
-17.6
0
17.6

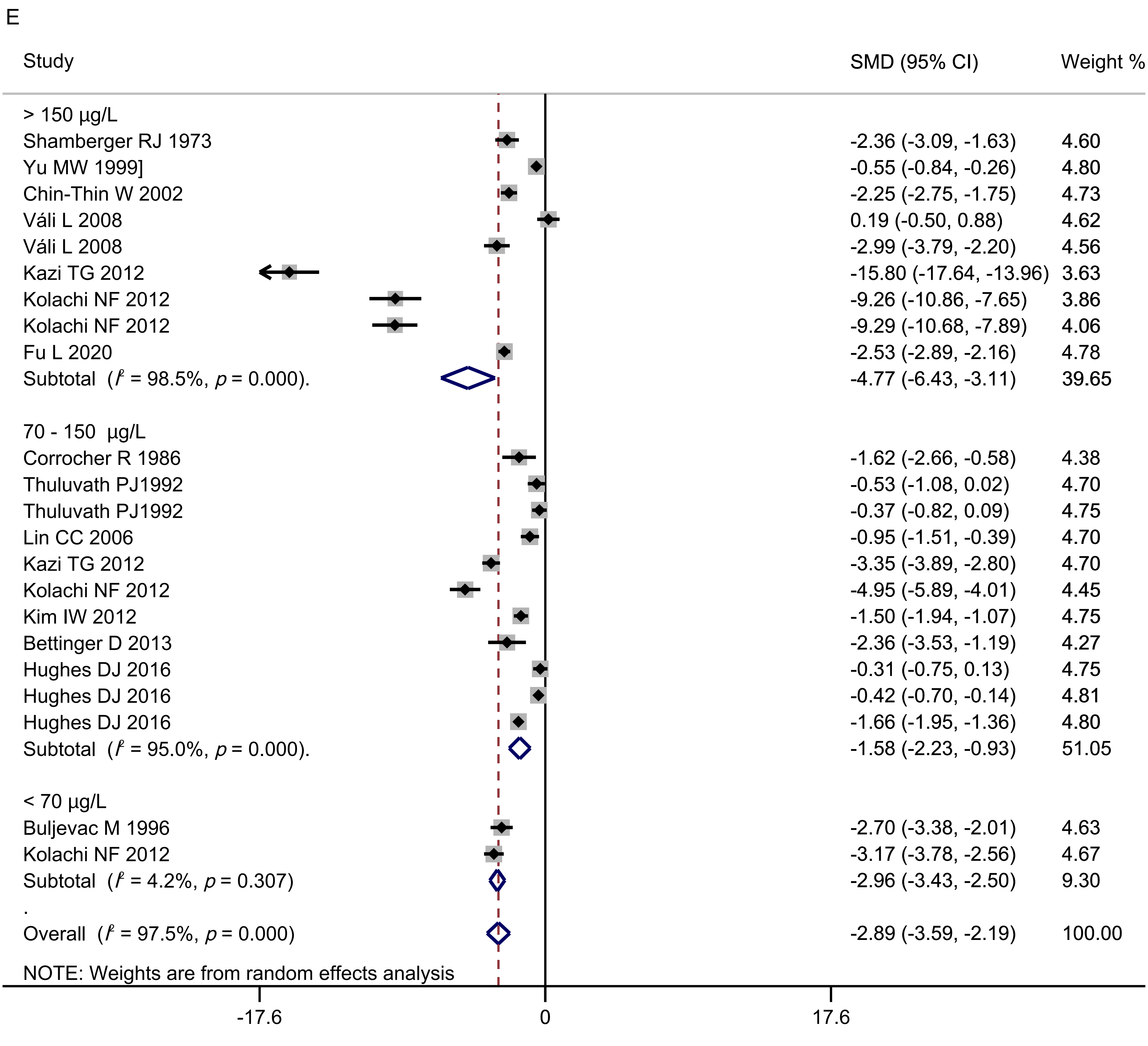

E
Study
SMD (95% CI)
Weight %
> 150 μg/L
Shamberger RJ 1973 -2.36 (-3.09, -1.63) 4.60
Yu MW 1999] -0.55 (-0.84, -0.26) 4.80
Chin-Thin W 2002 -2.25 (-2.75, -1.75) 4.73
Váli L 2008 0.19 (-0.50, 0.88) 4.62
Váli L 2008 -2.99 (-3.79, -2.20) 4.56
Kazi TG 2012 -15.80 (-17.64, -13.96) 3.63
Kolachi NF 2012 -9.26 (-10.86, -7.65) 3.86
Kolachi NF 2012 -9.29 (-10.68, -7.89) 4.06
Fu L 2020 -2.53 (-2.89, -2.16) 4.78
Subtotal (I^2 = 98.5%, p = 0.000). -4.77 (-6.43, -3.11) 39.65
70 - 150 μg/L
Corrocher R 1986 -1.62 (-2.66, -0.58) 4.38
Thuluvath PJ1992 -0.53 (-1.08, 0.02) 4.70
Thuluvath PJ1992 -0.37 (-0.82, 0.09) 4.75
Lin CC 2006 -0.95 (-1.51, -0.39) 4.70
Kazi TG 2012 -3.35 (-3.89, -2.80) 4.70
Kolachi NF 2012 -4.95 (-5.89, -4.01) 4.45
Kim IW 2012 -1.50 (-1.94, -1.07) 4.75
Bettinger D 2013 -2.36 (-3.53, -1.19) 4.27
Hughes DJ 2016 -0.31 (-0.75, 0.13) 4.75
Hughes DJ 2016 -0.42 (-0.70, -0.14) 4.81
Hughes DJ 2016 -1.66 (-1.95, -1.36) 4.80
Subtotal (I^2 = 95.0%, p = 0.000). -1.58 (-2.23, -0.93) 51.05
< 70 μg/L
Buljevac M 1996 -2.70 (-3.38, -2.01) 4.63
Kolachi NF 2012 -3.17 (-3.78, -2.56) 4.67
Subtotal (I^2 = 4.2%, p = 0.307) -2.96 (-3.43, -2.50) 9.30
.
Overall (I^2 = 97.5%, p = 0.000) -2.89 (-3.59, -2.19) 100.00
NOTE: Weights are from random effects analysis
-17.6
0
17.6

Figure S5. Forest plot in the meta-analysis of selenium levels and liver cancer. The dot represents the effect size of each study[13,35,40,44,48,50,59,64,65,71-76]. The line through the dot represents 95% CI of the effect size. The size of the square represents the weight, Wi. The broken line represents the effect size and the horizontal length of the diamond the 95% CI of the effect size for all studies combined. (A) stratified by study regions; (B) stratified by sample sources; (C) stratified by study design; (D) stratified by year of publication; (E) stratified by the reference blood (include whole blood, serum, plasma) selenium levels (70-150 μg/L)

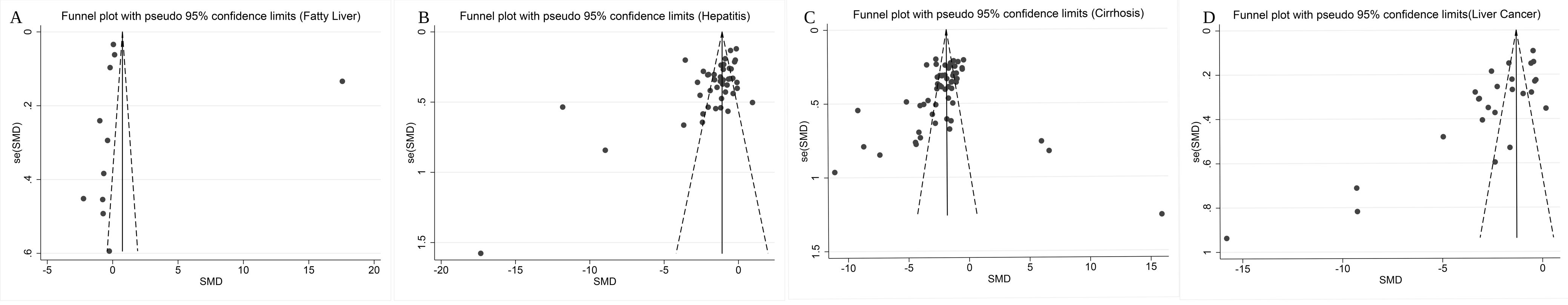
A
Funnel plot with pseudo 95% confidence limits (Fatty Liver)
se(SMD)
SMD
0
.2
.4
.6
-5
0
5
10
15
20
B
Funnel plot with pseudo 95% confidence limits (Hepatitis)
se(SMD)
SMD
0
.5
1
1.5
-20
-15
-10
-5
0
C
Funnel plot with pseudo 95% confidence limits (Cirrhosis)
se(SMD)
SMD
0
.5
1
1.5
-10
-5
0
5
10
15
D
Funnel plot with pseudo 95% confidence limits(Liver Cancer)
se(SMD)
SMD
0
.2
.4
.6
.8
1
-15
-10
-5
0

Figure S6. Funnel plot with pseudo 95% confidence limit of selenium levels and chronic liver diseases. Dotted lines represent pseudo 95% confidence intervals, dark spot represent studies, and continuous line represent estimated θ/v. (A) fatty liver diseases; (B) hepatitis; (C) liver cirrhosis; (D) liver cancer.

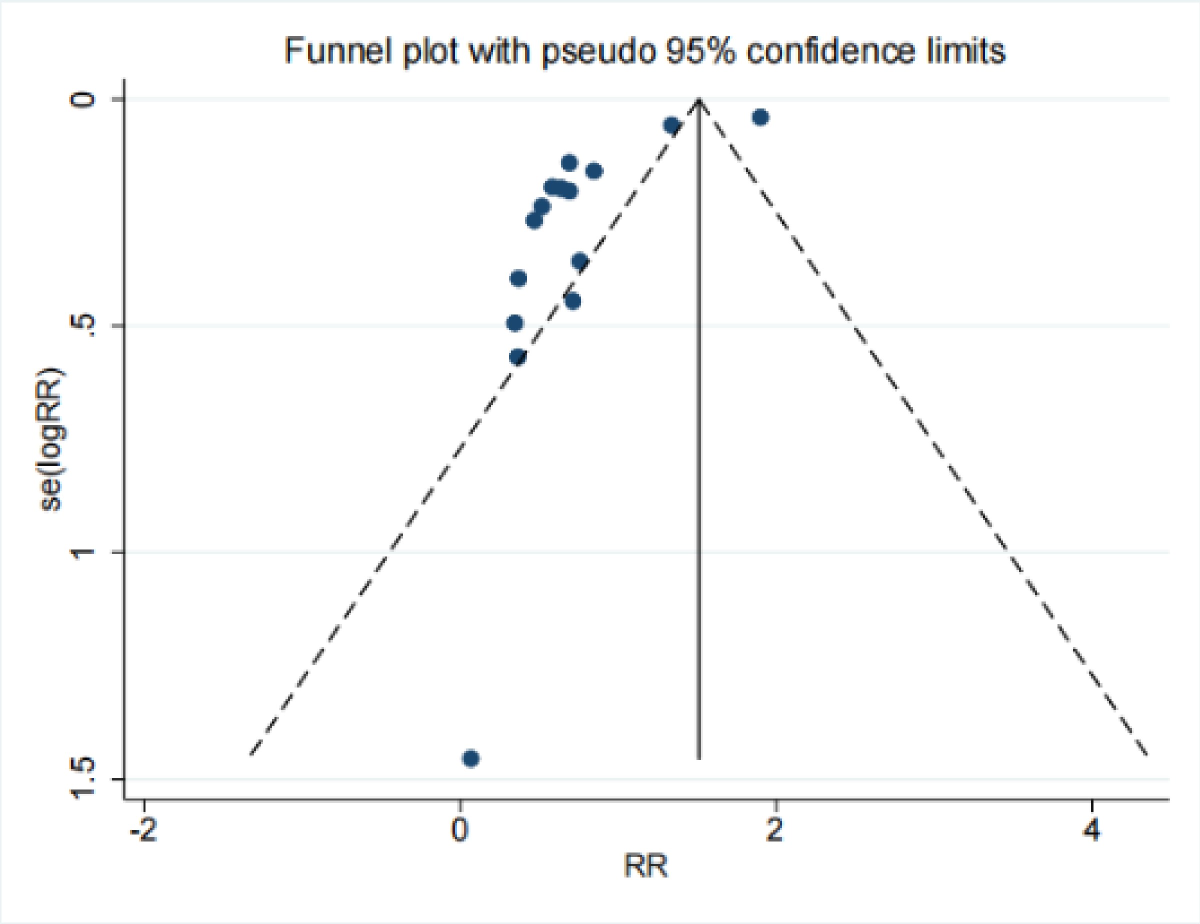

Figure S7. Funnel plot with pseudo 95% confidence limit of selenium intake and chronic liver diseases. Dotted lines represent pseudo 95% confidence intervals, dark spot represent studies, and continuous line represent estimated θ /v. selenium intake and chronic liver diseases.

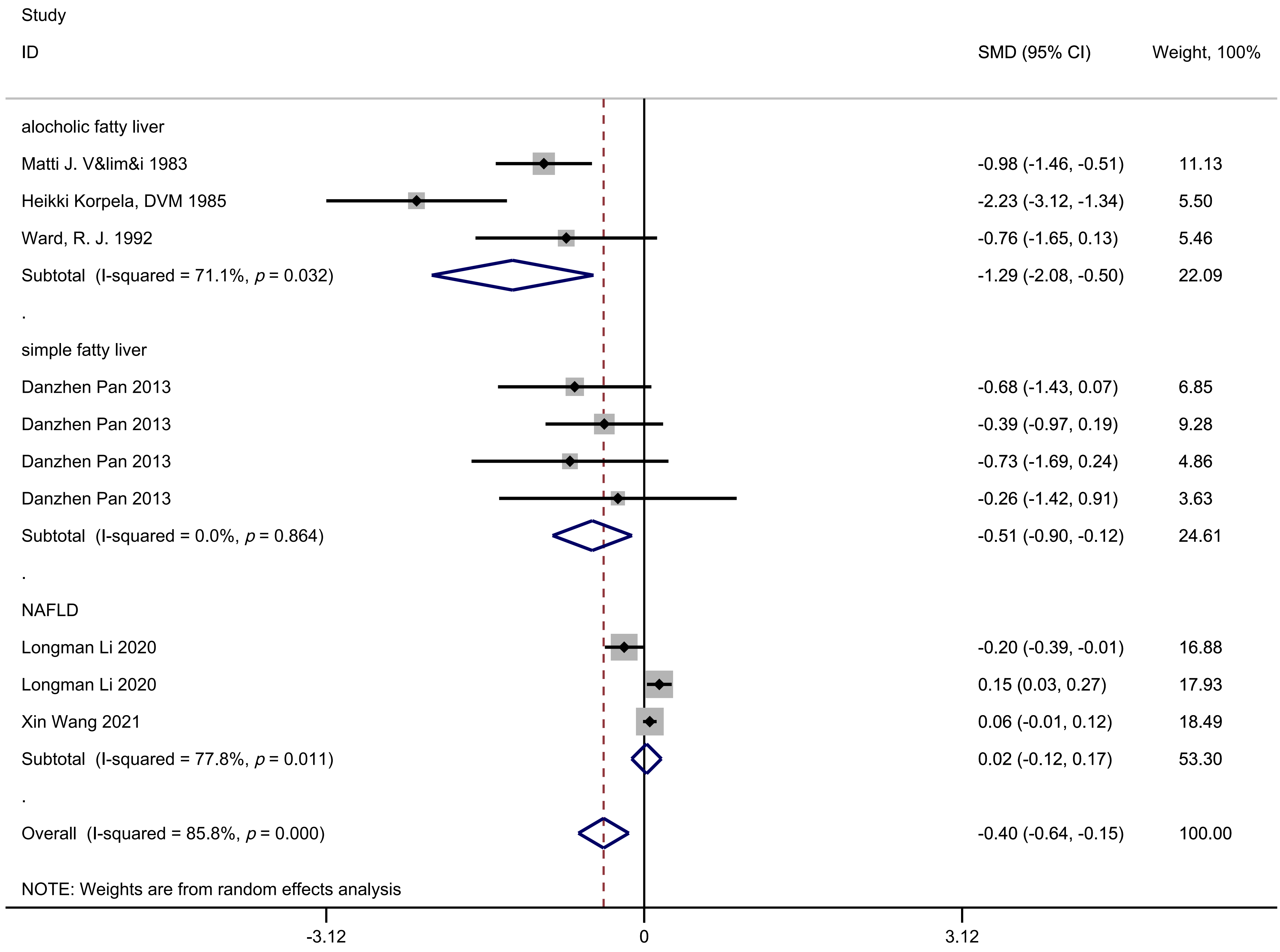
Study
ID
SMD (95% CI)
Weight, 100%
alocholic fatty liver
Matti J. V&lim&i 1983
-0.98 (-1.46, -0.51)
11.13
Heikki Korpela, DVM 1985
-2.23 (-3.12, -1.34)
5.50
Ward, R. J. 1992
-0.76 (-1.65, 0.13)
5.46
Subtotal (I-squared = 71.1%, p = 0.032)
-1.29 (-2.08, -0.50)
22.09
.
simple fatty liver
Danzhen Pan 2013
-0.68 (-1.43, 0.07)
6.85
Danzhen Pan 2013
-0.39 (-0.97, 0.19)
9.28
Danzhen Pan 2013
-0.73 (-1.69, 0.24)
4.86
Danzhen Pan 2013
-0.26 (-1.42, 0.91)
3.63
Subtotal (I-squared = 0.0%, p = 0.864)
-0.51 (-0.90, -0.12)
24.61
.
NAFLD
Longman Li 2020
-0.20 (-0.39, -0.01)
16.88
Longman Li 2020
0.15 (0.03, 0.27)
17.93
Xin Wang 2021
0.06 (-0.01, 0.12)
18.49
Subtotal (I-squared = 77.8%, p = 0.011)
0.02 (-0.12, 0.17)
53.30
.
Overall (I-squared = 85.8%, p = 0.000)
-0.40 (-0.64, -0.15)
100.00
NOTE: Weights are from random effects analysis
-3.12
0
3.12

Supplemental Figure 8. Forest plot in the meta-analysis of selenium levels and fatty liver diseases after removing the article on ultrasonic diagnosis. The dot represents the effect size of each study [16,30,32-34,77]. The line through the dot represents 95% CI of the effect size. The size of the square represents the weight, Wi. The broken line represents the effect size and the horizontal length of the diamond the 95% CI of the effect size for all studies combined.
