# Supplementary material for: Selenium Status in Patients with Chronic Liver Disease: A Systematic Review and Meta-Analysis
Source: Nutrients. 2022 Feb 23;14(5):952. doi: 10.3390/nu14050952 (PMC8912406; doi:10.3390/nu14050952)
Supplement: Supplementary file 1 [file nutrients-14-00952-s001.zip › Supplementary Table revised.pdf]

Supplementary Table S1. Initial Search Strategies

**Search 1: PubMed, search through April 28, 2021 (n= 1031)**
Search 1：((((selen*[Title/Abstract]) OR selenium [Title/Abstract]) OR selenium [MeSH Terms])) AND (((((liver [Title/Abstract]) OR hepat* [Title/Abstract]) OR liver diseases [Title/Abstract]) OR liver [MeSH Terms])) AND (((((((((epidemiology [Title/Abstract]) OR epidemiology*[MeSH Terms]) OR mortality* [Title/Abstract]) OR mortality*[MeSH Terms]) OR morbidity* [Title/Abstract]) OR morbidity[MeSH Terms]) OR incidence* [Title/Abstract]) OR incidence [MeSH Terms]))
**Search 2: Embase, search through April 28, 2021 (n= 1154)**
((('selenium':ab,ti) OR (selen*:ab,ti) OR ('selenium'/exp)) AND (('liver':ab,ti) OR (hepat*':ab,ti) OR ('liver diseases':ab,ti) OR ('liver'/exp))) AND ((('epidemiology':ab,ti) OR (mortality:ab,ti) OR ('epidemiology'/exp)) AND (('morbidity':ab,ti) AND ('incidence':ab,ti)
**Search 3: Cochrane Library, search through April 28, 2021 (n= 34)**
(((selenium):ti,ab,kw) OR ((selen*):ti,ab,kw) OR ([selenium] explode all trees)) AND (((liver):ti,ab,kw) OR ((liver diseases):ti,ab,kw) OR

((hepat*):ti,ab,kw) OR (([liver] explode all trees))) OR ((([epidemiology] explode all trees) OR (([mortality] explode all trees) OR ([morbidity] explode all trees)) AND (([incidence] explode all trees) OR ((epidemiology):ti,ab,kw) OR ((mortality):ti,ab,kw) OR (morbidity):ti,ab,kw)) OR (incidence):ti,ab,kw))
**Search 4: Web of science, search through April 28, 2021 (n= 4)**
((selen*) AND ((liver diseases OR (disease, liver) OR (hepat*) AND (epidemiology*) OR (mortality*) OR (morbidity*) OR (incidence*))

Supplementary Table S2. Characteristics of studies included in the meta-analysis

Study; Age group	Continent	Study Design	Sample Source	Case, n	Liver Progression	Mean ± SD	Control, n	Mean ± SD	Quality Score (NOS)
Shamberge 1973; Adult	USA	Case-control	Blood	14	Liver cancer	150 ± 26.3 µg/L	48	229 ± 35.2 µg/L	Serious (9)
				6	Other cirrhosis	136 ± 24.2 µg/L			
				3	Other hepatitis	145 ± 16.8 µg/L			
Burch 1978; Adult	USA	Case-control	Serum	8	Other cirrhosis	100 ± 9 µg/L	13	120 ± 6 µg/L	Serious (9)
Sullivan 1979; Adult	USA	Case-control	Serum	14	Other cirrhosis	90 ± 20 µg/L	37	120 ± 10 µg/L	Serious (9)
Aaseth 1980 ; Adult	Europe	Case-control	Serum	11	Alcoholic cirrhosis	50.5 ± 15.8 µg/L[1]	40	121 ± 19.7 µg/L[1]	Serious (8)
Aaseth 1982; Adult	Europe	Case-control	Serum	11	Alcoholic cirrhosis	95.6 ± 38.7 µg /L[1]	40	121 ± 19.7 µg/L[1]	Serious (9)
				12	Primary cirrhosis	108 ± 15.0 µg/L[1]			
				12	Other cirrhosis	96.3 ± 21.3 µg/L[1]			
				15	Viral hepatitis	88.4 ± 21.3 µgl/L[1] (HBV)			
				4	Viral hepatitis	94.8 ± 40.3 µg/L[1] (HCV)			
Välimäki 1983; Adult	Europe	Case-control	Serum	13	Alcoholic cirrhosis	43.3 ± 13.4 µg/L	45	83.1 ± 15.7 µg/L	Serious (9)
				17	Primary cirrhosis	47.3 ± 7.9 µg/L			
Korpela 1985; Adult	Europe	Case-control	Serum	11	Alcoholic fatty liver disease	55.8 ± 21.2 µg/L	25	88.7 ± 11.0 µg/L	Serious (9)
				6	Alcoholic hepatitis	46.0 ± 14.1 µg/L			
				22	Alcoholic cirrhosis	41.1 ± 12.8 µg/L			

Milman 1986; Adult	Europe	Case-control	Liver	14	Alcoholic cirrhosis	316 ± 158 μg/kg[1]	74	2210 ± 395 μg/kg[1]	Serious (9)
				13	Primary cirrhosis	8690 ± 474 μg/kg[1]			
Sharda 1986; Children	Asia	Case-control	Liver	20	Other cirrhosis	160 ± 10 μg/kg[1]	20	100 ± 10 μg/kg[1]	Serious (9)
			Hair	20	Other cirrhosis	400 ± 80 μg/kg[1]	20	500 ± 60 μg/kg[1]	
			Nail	20	Other cirrhosis	1000 ± 40 μg/kg[1]	20	700 ± 50 μg/kg[1]	
Johansson 1986; Adult	Europe	Case-control	Plasma	21	Alcoholic cirrhosis	58.4 ± 11.1 μg/L[1]	16	79.8 ± 11.1 μg/L[1]	Serious (1)
			Blood	21	Alcoholic cirrhosis	68.7 ± 12.6 μg/L[1]	16	94.0 ± 18.2 μg/L[1]	
			Plasma	15	Alcoholic hepatitis	78.2 ± 19.7 μg/L[1]	16	79.8 ± 11.1 μg/L[1]	
Corrocher 1986; Adult	Europe	Case-control	Serum	12	Liver cancer	84.5 ± 32.4 μg/L[1]	8	126.4 ± 8.7 μg/L[1]	Serious (9)
Dworkin 1988; Adult	USA	Case-control	liver	12	Alcoholic cirrhosis	731 ± 77 μg/kg[1]	13	1310 ± 166 μg/kg[1]	Serious (8)
Casaril 1989; Adult	Europe	Case-control	Serum	55	Other cirrhosis	45.0 ± 15.8 μg/L[1]	47	72.7 ± 12.6 μg/L[1]	Serious (9)
Välimäki 1991; Adult	Europe	Case-control	Blood	8	Primary cirrhosis	288 ± 15.0 μg/L[1]	8	318 ± 17.4 μg/L[1]	Serious (9)
Ward 1992; Adult	Europe	Case-control	Plasma	11	Alcoholic fatty liver disease	77.4 ± 49.0 μg/L[1]	10	109.9 ± 34.0 μg/L[1]	Serious (7)
				5	Alcoholic cirrhosis	62.4 ± 29.2 μg/L[1] (P-Ch A-B)			
				4	Alcoholic cirrhosis	61.6 ± 16.6 μg/L[1] P-Ch C)			
Thuluvath 1992; Adult	Europe	Case-control	Serum	51	Alcoholic cirrhosis	63.6 ± 18.2 μg/L	56	87.8 ± 21.1 μg/L	Serious (9)
				45	Primary cirrhosis	60.6 ± 13.6 μg/L			
				8	Other cirrhosis	59.3 ± 13.3 μg/L			

				19	Other hepatitis	73.9 ± 20.3 μg/L			
				9	Viral hepatitis	65.4 ± 19.0 μg/L			
				17	Liver cancer	76.8 ± 18.3 μg/L			
Gerli 1992; Adult	Europe	Case-control	Serum	21	Alcoholic hepatitis	67.1 ± 18.2 μg/L[1] (	50	75.0 ± 15.0 μg/L[1]	Serious (9)
				22	Alcoholic cirrhosis	67.1 ± 15.8 μg/L[1] (P-Ch A)			
				30	Alcoholic cirrhosis	56.1 ± 19.7 μg/L[1] (P-Ch B)			
				21	Alcoholic cirrhosis	48.2 ± 10.3 μg/L[1] (P-Ch C)			
Buljevac 1996; Adult	Europe	Case-control	Serum	49	Liver cirrhosis	39.0± 14.0 μg/L[1]	202	66.8 ± 9.13 μg/L[1]	Serious (7)
				10	Liver cancer	42.0 ± 10.6 μg/L[1]			
Loguercio 1997; Adult	Europe	Case-control	Plasma	8	Viral hepatitis	120 ± 30 μg/L[1]	10	100 ± 10 μg/L	Moderate (6)
				18	Primary cirrhosis	70 ± 10 μg/L[1] malnourished			
				18	Primary cirrhosis	70 ± 10 μg/L[1] malnourished			
			Blood	8	Viral hepatitis	140 ± 20 μg/L[1]			
				18	Primary cirrhosis	100 ± 10 μg/L[1] malnourished	10	180 ± 30 μg/L[1]	
				14	Primary cirrhosis	90 ± 10 μg/L[1] Well-nourished			
Guarini 1998; Adult	Europe	Case-control	Serum	20	Alcoholic cirrhosis	75.0 ± 15.0 μg/L[1]	40	94.0 ± 15.8 μg/L[1]	Serious (9)
				13	Other cirrhosis	75.8 ± 27.6 μg/L[1]			
Yu 1999; Adult	Asia	Cohort study	Plasma	139	Viral hepatitis	145 ± 37.6 μg/L	138	151 ± 35.2 μg/L	Serious (9)
				69	Liver cancer	132± 30.9 μg/L			

Navarro-Alarcón 2002; Adult	Europe	Cross-section	Serum	12	Other cirrhosis	41.0 ± 12.4 μg/L	130	74.9 ± 27.3 μg/L	Serious[3] (7)
				38	Viral hepatitis	52.4 ± 15.6 μg/L			
Chin-Thin 2002; Adult	Asia	Case-control	Blood	51	Liver cancer	180 ± 20 μg/L[1]	50	280 ± 60 μg/L[1]	Serious (9)
Czuczejko 2003; Adult	Europe	Case-control	Blood	23	Viral hepatitis	58.6 ± 13.3 μg/L[1] (ALT ≤ 40 U/L)	50	89.5 ± 15.6 μg/L[1]	Serious (9)
				36	Viral hepatitis	74.3 ±15.0 μg/L[1] (ALT ≥ 40 U/L)			
			Plasma	23	Viral hepatitis	43.6 ± 12.1 μg/L[1] (ALT ≤ 40 U/L)	50	67.4 ± 11.7 μg/L[1]	
				36	Viral hepatitis	53.7 ± 11.6 μg/L[1] (ALT ≥ 40 U/L)			
			Blood	26	Other cirrhosis	52.5 ± 15.7 μg/L[1] (ALT ≤ 40 U/L)	50	89.5 ± 15.6 μg/L[1]	
				38	Other cirrhosis	64.4 ± 17.5 μg/L[1] (ALT ≥ 40 U/L)			
			Plasma	26	Other cirrhosis	37.4 ± 11.6 μg/L[1] (ALT ≤ 40 U/L)	50	67.4 ± 11.7 μg/L[1]	
				38	Other cirrhosis	48.7 ± 15.0 μg/L[1] (ALT ≥ 40 U/L)			
Sakoda 2005; Adult	Asia	Nest case control	Nail	166	Liver cancer	3.1 ± 1.5 ppm	394	3.5 ± 0.45 ppm	Serious (10)
Pemberton 2005; Adult	Europe	Case-control	Serum	24	Alcoholic cirrhosis	46.2 ± 7.2 μg/L[1]	49	101 ± 3.28 μg/L[1]	Serious (9)
Ko 2005; Adult	Asia	Cohort study	Plasma	33	Viral hepatitis	159 ± 5.3 μg/L	31	217 ± 7.44 μg/L	Serious

			Blood	33	Viral hepatitis	55.1 ± 3.79 μg/L	31	139 ± 5.76 μg/L	(9)
Sayed 2005; Adult	Africa	Cross-section	Blood	25	Viral hepatitis	154 ± 12.8 μg/L[1] (HBsAg positive)	41	166 ± 23.5 μg/L[1]	Serious[3] (8)
				12	Viral hepatitis	154 ± 12.1 μg/L[1] (Anti-HCV Ab positive)			
				6	Viral hepatitis	158 ± 6.6 μg/L[1] (Combined infection)			
Lin 2006; Adult	Asia	Case-control	Blood	40	Viral hepatitis	124 ± 20.4 μg/L (HBV carriers)	50	129 ± 21.5 μg/L	Serious (8)
				50	Viral hepatitis	124 ± 22.6 μg/L (HBV)			
				18	Liver cancer	109 ± 21.8 μg/L			
				20	Other cirrhosis	118 ± 25.3 μg/L			
Váli 2008; Adult	Europe	Case-control	Blood	11	Liver cancer	314 ± 83.7 μg/kg (Benign)	30	301 ± 57.2 μg/kg	Serious (8)
				23	Liver cancer	129 ± 58.3 μg/kg (HCC)			
González-Reimers 2009; Adult	Europe	Case-control	Serum	9	Viral hepatitis	81.3 ± 10.1 μg/L (HCV)	20	82.1 ± 9.1 μg/L	Serious (9)
				12	Viral hepatitis	73.4 ± 14.5 μg/L (HCV patient drink)			
				19	Alcoholic hepatitis	70.0 ± 14.0 μg/L			
Balamtekin 2010; children	Asia	RCT	Plasma	35	Viral hepatitis	18.4 ± 9.06 μg/L[1]	27	44.7 ± 10.2 μg/L[1]	Serious[2] (9)
Martínez-Peinado 2010; Adult	Europe	Case-control	Blood	93	Other cirrhosis	56.9 ± 18.9 μg/L[1]	30	73.1 ± 19.0 μg/L[1]	Serious (9)
Uslu 2010; children	Asia	Case-control	Serum	38	Other cirrhosis	42.4 ± 8.2 μg/L	41	64.4 ± 16.9 μg/L	Serious (7)

Himoto 2011; adult	Asia	Case-control	Serum	10	Viral hepatitis	125 ± 24 µg/L[1] (HCV F3)	11	148 ± 16 µg/L[1]	Serious (9)
				10	Viral hepatitis	113 ± 13 µg/L[1] (HCV F4)			
Khan 2012; Adult	Asia	Case-control	Blood	5	Viral hepatitis	138 ± 0.36 µg/L (HBV female)	10	199 ± 104 µg/L	Serious (9)
				36	Viral hepatitis	77.4 ± 0.47 µg/L (HBV male)			
				38	Viral hepatitis	107.5 ± 0.44 µg/L (HCV female)	16	214 ± 164 µg/L	
				71	Viral hepatitis	101.6 ± 0.55 µg/L (HCV male)			
Kolachi 2012; Adult	Asia	RCT	Hair	26	Other cirrhosis	680 ± 310 µg/kg[1] (20-45 years old)	43	1280 ± 250 µg/kg[1]	Serious[2] (4)
				52	Liver cancer	660 ± 140 µg/kg[1] (20-45 years old)			
				14	Other cirrhosis	710 ± 410 µg/kg[1] (46-60 years old)	32	1290 ± 430 µg/kg[1]	
				40	Liver cancer	690 ± 380 µg/kg[1] (46-60 years old)			
			Serum	26	Other cirrhosis	48.5 ± 6.90 µg/L (20-45 years old)	43	69.6 ± 11.2 µg/L	
				52	Liver cancer	44.8 ± 2.84 µg/L (20-45 years old)			
				14	Other cirrhosis	45.8 ± 8.90 µg/L (46-60 years old)	32	75.7 ± 8.90 µg/L	
				40	Liver cancer	43.1 ± 3.85 µg/L (46-60			

						years old)			
			Blood	26	Other cirrhosis	114 ± 6.32 μg/L (20-45 years old)	43	175 ± 7.41 μg/L	
				52	Liver cancer	104 ± 7.83 μg/L (20-45 years old)			
				14	Other cirrhosis	99.8 ± 10.2 μg/L (46-60 years old)	32	163 ± 7.82 μg/L	
				40	Liver cancer	97.7 ± 6.38 μg/L (46-60 years old)			
Kazi 2012; adult	Asia	RCT	Serum	31	Liver cancer	36.2 ± 3.95 μg/L	120	77.0 ± 13.5 μg/L	Serious[2] (4)
				41	Other cirrhosis	41.8 ± 12.5 μg/L			
			Blood	31	Liver cancer	63.5 ± 9.22 μg/L	120	180 ± 6.83 μg/L	
				41	Other cirrhosis	112 ± 8.97 μg/L			
Bettinger 2013; Adult	Europe	Case-control	Plasma	12	Viral hepatitis	84.7 ± 16.4 μg/L	10	118 ± 15.7 μg/L	Serious (9)
				10	Liver cancer	85 ± 11.5 μg/L			
				10	Other cirrhosis	99.8 ± 11 μg/L			
Pan 2013; Adult	Asia	Case-control	Hair	15	Alcoholic hepatitis	450 ± 130 μg/kg[1] (15-25 years old)	18	680 ± 180 μg/kg[1]	Serious (9)
				12	Simple fatty liver disease	560 ± 170 μg/kg[1] (15-25 years old)			
				30	Alcoholic hepatitis	470 ± 130 μg/kg[1] (26-35 years old)	34	620 ± 160 μg/kg[1]	
				18	Simple fatty liver disease	560 ± 140 μg/kg[1](26-35 years old)			

				18	Alcoholic hepatitis	310 ± 90 µg/kg[1] (36-45 years old)	16	500 ± 110 µg/kg[1]	
				6	Simple fatty liver disease	420 ± 110 µg/kg[1] (36-45 years old)			
				15	Alcoholic hepatitis	340 ± 80 µg/kg[1] (46-55 years old)	10	410 ± 80 µg/kg[1]	
				4	Simple fatty liver disease	390 ± 70 µg/kg[1] (46-55 years old)			
Kolachi 2013; Adult	Asia	RCT	Blood	132	Viral hepatitis	107 ± 5.56 µg/L (Certified Value)	128	172 ± 5.43 µg/L	Serious[2] (4)
			Serum	132	Viral hepatitis	51.6 ± 2.66 µg/L (Measured Value)	128	68.4 ± 6.12 µg/L	
Nangliya 2015; Adult	Asia	Cross-section	Serum	150	Other cirrhosis	70.8 ± 15.3 µg/L[1]	50	124.7 ± 16.6 µg/L[1]	Serious[3] (7)
Hughes 2016; Adult	Europe	Cohort study	Blood	40	Liver cancer	82.3 ± 18.3 µg/L (HCC)	40	87.5 ± 15.1 µg/L	Serious (9)
				100	Liver cancer	82.1 ± 8.17 µg/L (GBTC)	100	85.9 ± 9.77 µg/L	
				121	Liver cancer	71.3 ± 5.77 µg/L (IHBC)	121	85.2 ± 10.4 µg/L	
Yang 2016; Adult	Asia	Cross-section	Blood	3732	NAFLD	270 ± 5.35 µg/L	5118	192 ± 3.60 µg/L	Serious[3] (8)
Prystupa 2017; Adult	Europe	case-control	Blood	29	Alcoholic cirrhosis	68 ± 14 µg/L[1] (P-Ch A)	20	100 ± 13 µg/L[1]	Serious (9)
				36	Alcoholic cirrhosis	85 ± 16 µg/L[1] (P-Ch B)			
				34	Alcoholic cirrhosis	72 ± 17 µg/L[1](P-Ch C)			
Dhanda 2020; Adult	Europe	Cohort study	Serum	302	Alcoholic hepatitis	47.4 ± 23.7 µg/L[1]	15	103 ± 15.8 µg/L[1]	Serious (9)
				11	Other cirrhosis	63.2 ± 31.6 µg/L[1]			

Fu 2020; Adult	Asia	Case-control	Serum	105	Liver cancer	88.3 ± 20.6 µg/L	105	167 ± 38.9 µg/L	Serious (9)
Li 2020; Adult	Asia	Cross-section	Blood	144	NAFLD	157 ± 6.95 µg/L (from Longitudinal analysis)	522	159 ± 8.33 µg/L	Serious[3] (7)
				330	NAFLD	157 ± 8.04 µg/L (from Cross-sectional analysis)	1264	156 ± 7.69 µg/L	
Wang 2021; Adult	USA	Cross-section	Serum	1319	NAFLD	129 ± 40.0 µg/L	2508	127 ± 40.0 µg/L	Serious[3] (8)

NAFLD: non-alcoholic fatty liver, HBV: hepatitis B virus, HCV: hepatitis C virus, SD: standard deviation, HCC: Hepatocellular carcinoma ; GBTC: Biliary tract cancer, IHBC:Intrahepatic bile duct cancer, P-Ch: Child-Pugh , ALT: alanine transaminase,

[1] the data were converted to µg/L or µg/kg for consistency.

[2] the quality score measured by Jadad scale, the score ≥ 4 means that the quality of this study is serious

[3] the quality score measured by AHRQ scale, the score ≥ 7 means that the quality of this study is serious
